# Interleukin‐2/anti‐interleukin‐2 immune complex attenuates cold ischemia‐reperfusion injury after kidney transplantation by increasing renal regulatory T cells

**DOI:** 10.1002/ctm2.1631

**Published:** 2024-03-19

**Authors:** Joon Young Jang, Hyung Woo Kim, Ji‐Jing Yan, Tae Kyeom Kang, Wook‐Bin Lee, Beom Seok Kim, Jaeseok Yang

**Affiliations:** ^1^ Department of Internal Medicine Yonsei University College of Medicine Seoul Republic of Korea; ^2^ Natural Product Research Center Korea Institute of Science and Technology Gangneung Republic of Korea

**Keywords:** cold ischemia‐reperfusion injury, IL‐2/anti‐IL‐2 immune complex, kidney transplantation, regulatory T cells

## Abstract

**Background:**

Cold ischemia‐reperfusion injury (IRI) is an unavoidable complication of kidney transplantation. We investigated the role of regulatory T cells (Treg) in cold IRI and whether the interleukin (IL)‐2/anti‐IL‐2 antibody complex (IL‐2C) can ameliorate cold IRI.

**Methods:**

We developed a cold IRI mouse model using kidney transplantation and analyzed the IL‐2C impact on cold IRI in acute, subacute and chronic phases.

**Results:**

Treg transfer attenuated cold IRI, while Treg depletion aggravated cold IRI. Next, IL‐2C administration prior to IRI mitigated acute renal function decline, renal tissue damage and apoptosis and inhibited infiltration of effector cells into kidneys and pro‐inflammatory cytokine expression on day 1 after IRI. On day 7 after IRI, IL‐2C promoted renal regeneration and reduced subacute renal damage. Furthermore, on day 28 following IRI, IL‐2C inhibited chronic fibrosis. IL‐2C decreased reactive oxygen species‐mediated injury and improved antioxidant function. When IL‐2C was administered following IRI, it also increased renal regeneration with Treg infiltration and suppressed renal fibrosis. In contrast, Treg depletion in the presence of IL‐2C eliminated the positive effects of IL‐2C on IRI.

**Conclusion:**

Tregs protect kidneys from cold IRI and IL‐2C inhibited cold IRI by increasing the renal Tregs, suggesting a potential of IL‐2C in treating cold IRI.

**Key Points:**

- Interleukin (IL)‐2/anti‐IL‐2 antibody complex attenuated acute renal injury, facilitated subacute renal regeneration and suppressed chronic renal fibrosis after cold ischemia‐reperfusion injury (IRI) by increasing the renal Tregs.
- IL‐2/anti‐IL‐2 antibody complex decreased reactive oxygen species‐mediated injury and improved antioxidant function.
- This study suggests the therapeutic potential of the IL‐2/anti‐IL‐2 antibody complex in kidney transplantation‐associated cold IR.

## Graphical abstract

Interleukin (IL)‐2/anti‐IL‐2 antibody complex attenuated acute renal injury, facilitated subacute renal regeneration and suppressed chronic renal fibrosis after cold ischemia‐reperfusion injury (IRI) by increasing the renal Tregs. IL‐2/anti‐IL‐2 antibody complex decreased reactive oxygen species‐mediated injury and improved antioxidant function. This study suggests the therapeutic potential of the IL‐2/anti‐IL‐2 antibody complex in kidney transplantation‐associated cold IRI.

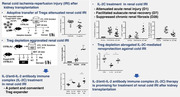

## 1 INTRODUCTION

Kidney transplantation is the preferred treatment for patients with end‐stage kidney disease. Renal ischemia‐reperfusion injury (IRI) is an unavoidable adverse event that occurs after kidney transplantation, particularly in kidney transplantation from deceased donors.^1^ IRI is caused by the interruption of blood supply and hypoxia, which is followed by subsequent restoration of blood supply and reoxygenation. Renal ischemia induces mitochondrial dysfunction, acute inflammation, endothelial dysfunction and sublethal tubular epithelial injury and subsequent reperfusion amplifies tissue injury by increasing chemokine, cytokine and reactive oxygen species (ROS) production.^1^, ^2^, ^3^ ROS including superoxide, hydrogen peroxide and hypochlorous acid, mediate renal IRI by activation of inflammatory responses.^1^, ^2^, ^4^, ^5^, ^6^ Re‐oxygenation increases ROS in the parenchymal, endothelial and infiltrating lymphocytes and ROS production is stimulated by neutrophils in IRI.^2^ IRI has recently been considered an acute inflammatory response followed by chronic fibrosis.^7^

Renal IRI in kidney transplantation exhibits a special form of IRI–cold IRI–which is characterized by discontinuation of blood supply (warm ischemia), cold storage (cold ischemia) and reperfusion. Cold IRI is associated with the cold storage of procured donor kidneys in contrast to conventional “warm IRI.” Although cold storage is used to slow anaerobic metabolism and the subsequent accumulation of metabolic waste products with adenosine triphosphate depletion, it cannot prevent ischemic damage. In addition, it seriously damages organs coupled with direct injury from hypothermia.^4^, ^8^ Cold IRI, with additional damage during cold ischemia, induces more severe injury than that caused by warm IRI.^9^

Both innate and adaptive immune cells are involved in the injury and recovery processes of renal IRI from the acute injury phase to subacute recovery and chronic fibrosis phases.^7^ The dynamic balance between immune effector and suppressor cells influences the course of renal warm IRI.^10^, ^11^, ^12^, ^13^, ^14^, ^15^, ^16^, ^17^, ^18^, ^19^ Notably, regulatory T cells (Tregs) play a crucial role in the renal warm IRI not associated with kidney transplantation by suppressing acute injury and facilitating recovery after IRI.^10^, ^11^ Interestingly, previous studies demonstrated that immune complex (IL‐2C) therapy of interleukin (IL)−2 and a particular anti‐IL‐2 monoclonal antibody (JES6‐1) preferentially induces the expansion of Tregs with a minimal proliferation of effector T and natural killer (NK) cells.^20^, ^21^, ^22^ Furthermore, IL‐2C therapy ameliorates renal warm IRI.^12^ Immune cells also play important roles in cold IRI as well as warm IRI.^23^ However, the role of Tregs in cold IRI has not been previously studied, although IRI in human kidney transplantation is cold IRI rather than warm IRI. Moreover, it is unclear whether convenient IL‐2C therapy can attenuate a relatively severe cold IRI by expanding Tregs. Here, the role of Tregs in renal cold IRI was investigated using a mouse kidney transplantation model and determined whether IL‐2C therapy can mitigate cold IRI.

## 2 MATERIALS AND METHODS

### 2.1 Animals and materials

C57BL6/J mice (B6, 10–12 weeks; male; 27–30 g), forkhead box P3 (Foxp3)‐green fluorescent protein (GFP)‐diphtheria toxin receptor (DTR) B6 mice and Foxp3‐knock‐in (KI) B6 mice were used in all experiments. All experiments were approved by the Institutional Animal Care and Use Committee of the Yonsei University Health System (IACUC 2021−0131) and adhered to the NIH Guide for the Care and Use of Laboratory Animals or the equivalent. Recombinant mouse IL‐2 and anti‐mouse IL‐2 monoclonal antibodies (JES6‐1) were provided from BioLegend and BioXcel, respectively. IL‐2C was prepared by mixing IL‐2 (1 µg) with anti‐IL‐2 antibodies (5 µg) at a ratio of 1:5 and incubating at 37°C for 30 min, according to optimized protocols established by the previous studies.^12^, ^20^, ^21^, ^22^ Daily dose of IL‐2C was 6 µg in all experiments. DT (1 µg; Merck) was intraperitoneally administered twice to deplete Tregs in Foxp3‐GFP‐DTR B6 mice.

### 2.2 Development of mouse models of kidney transplantation and renal cold IRI

The right kidney from donor mice was transplanted into syngeneic recipients simultaneously with bilateral native nephrectomy by following previously described procedures with modifications.^24^, ^25^ The donor kidneys were flushed with cold heparinized histidine‐tryptophan‐ketoglutarate (HTK) solution (Custodiol; Dr. Franz Köhler Chemie GmbH) and then kept in HTK solution in an ice‐water bath for the indicated time (cold ischemic time, CIT) to induce cold IRI. The warm ischemic time (WIT) from taking out donor kidneys from cold storage to reperfusion after graft anastomosis was approximately 24 min. The kidney graft functions were monitored by measuring whole blood creatinine and blood urea nitrogen (BUN, upper detection limit of 140 mg/dL) levels using CHEM8^+^ cartridges and an i‐STAT Analyzer (Abbott).^26^, ^27^

### 2.3 Isolation and adoptive transfer of Tregs

IL‐2C was intraperitoneally administered to Foxp3‐GFP‐KI mice for five consecutive days up to 1 day before harvesting cells. Splenic CD4^+^ T cells were isolated by a MojoSort Isolation Kit (BioLegend). GFP^+^ cells representing CD4^+^Foxp3^+^ Tregs were isolated using the FACSAria II cell sorter (BD Biosciences). Tregs were intravenously transferred to recipient mice 1 day prior to kidney transplantation. In vitro suppressive activity of sorted Tregs against T cell proliferation was also assessed using a suppression assay and its detailed procedure is described in the Supporting Information.

### 2.4 Flow cytometric analysis and enzyme‐linked immunosorbent assay

Kidneys were procured after perfusion, and renal leukocytes were prepared using a Stomacher 80 Biomaster (Seward), as previously described.^17^, ^28^ Staining and flow‐cytometric analytic methods for renal cells are provided in the Supporting Information and Table S1. The gating strategy is summarized in Figure S1. Systemic cytokine levels were measured using enzyme‐linked immunosorbent assay (BioLegend).

### 2.5 Measurement of 8‐hydroxy‐2′‐deoxyguanosine, malondialdehyde and glutathione levels and determination of superoxide dismutase activity

The oxidative biochemical parameters such as 8‐hydroxy‐2′‐deoxyguanosine (8‐OhdG), malondialdehyde (MDA), glutathione (GSH) and superoxide dismutase (SOD) activity were measured in plasma and renal tissues. The detailed procedure is described in the Supporting Information section.

### 2.6 Western blotting and real‐time polymerase chain reaction

The detailed procedures are found in the Supporting Information. The antibodies and primer information are provided in Tables S1 and S2, respectively. Full blot images are shown in Figure S2.

### 2.7 Histological analysis

Renal apoptosis, renal regeneration and renal fibrosis were assessed by performing dUTP‐biotin nick‐end labelling (TUNEL; Abcam), Ki67, aquaporin‐1 (AQP‐1), vascular endothelial cell growth factor (VEGF) and Masson trichrome (MT) staining, respectively. The tubular injury score was measured in periodic acid‐Schiff (PAS)‐stained sections.^29^ Immunohistochemical staining was performed for α‐smooth muscle actin (α‐SMA) and E‐cadherin to assess renal epithelial‐to‐mesenchymal transition and fibrosis. Immunofluorescence (IF) staining for dihydroethidium (DHE) was performed to quantify the amount of ROS contents in renal tissues. IF staining was also performed for F4/80^+^ macrophages, Gr‐1^+^ neutrophils and CD4^+^Foxp3^+^ Tregs. The detailed procedures and analytic methods of PAS, TUNEL, Ki67, AQP‐1, VEGF, MT and IF staining are described in the Supporting Information.

### 2.8 Statistical analysis

Data are presented as the mean ± standard error of the mean. Continuous variables were compared by Student's *t*‐test. Non‐parametric tests, such as the Mann‐Whitney test, were used when data were not normally distributed. Kaplan–Meier analysis was used to assess survival rates, and a log‐rank test was used for survival comparison. Values of *p* < .05 were considered statistically significant. Analyses were performed using GraphPad Prism (version 7.0; GraphPad Software).

## 3 RESULTS

### 3.1 Cold IRI with a long CIT induced a relatively severe renal injury

**FIGURE 1:**
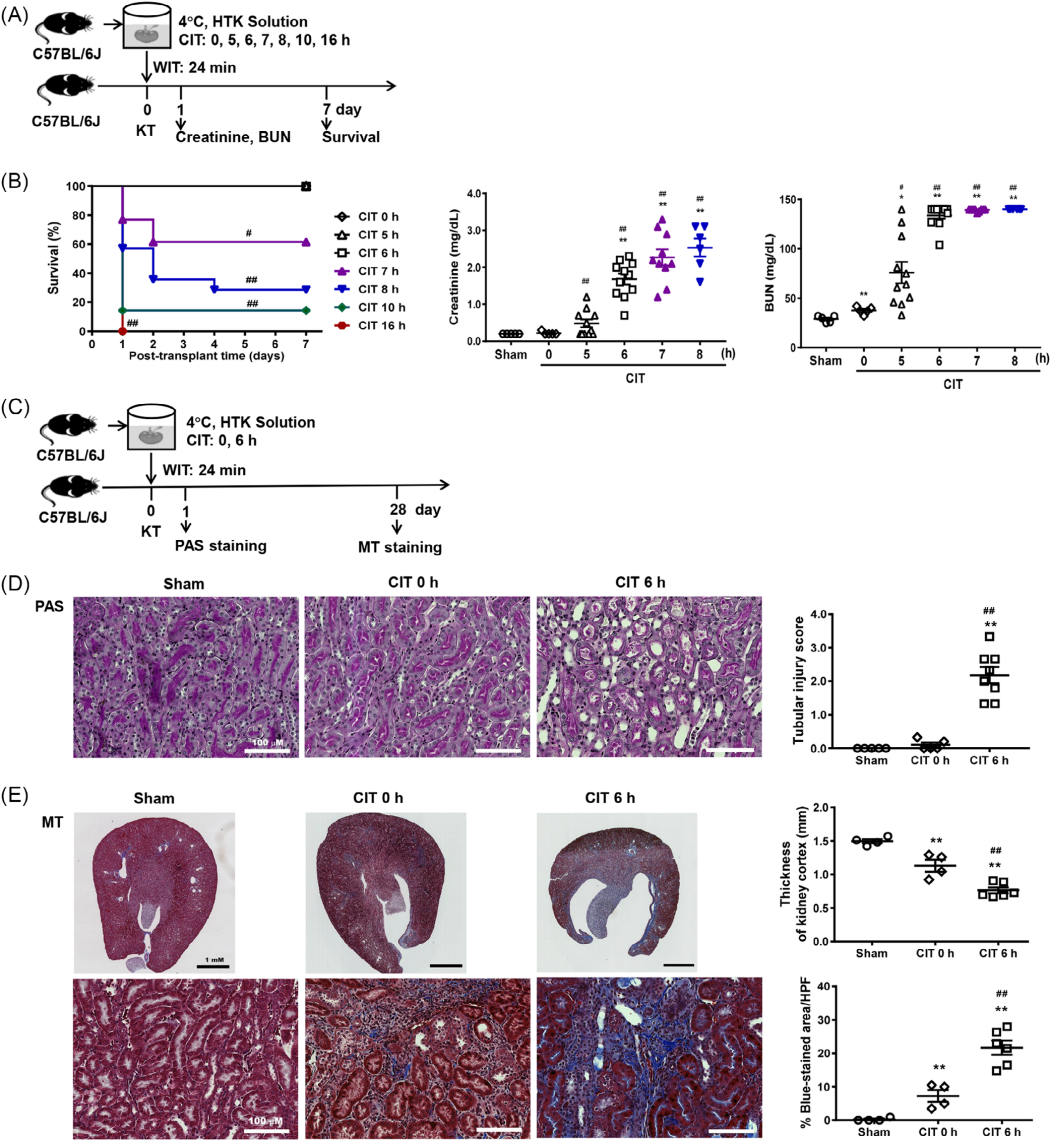
Cold IRI with a long CIT induced relatively severe renal injury. (A) For renal cold IRI, syngeneic mouse kidney transplantation was performed with a fixed WIT of 24 min and variable CIT of 0, 5, 6, 7, 8, 10 or 16 h. (B) Mouse mortality was assessed according to CIT. Renal functions on day 1 were assessed by measuring blood creatinine and BUN levels. (C) Renal tissues were harvested on days 1 and 28 after IRI (CIT, 0 or 6 h). (D) Renal tissue injury scores based on PAS staining on day 1. Magnification, 200×. (E) Renal cortical thickness (magnification, 40×; upper panels) and fibrosis (magnification, 200×; lower panels) based on MT staining on day 28. Lines and whiskers in dot plots indicate the mean and SEM, respectively. *p < .05, **p < .01 compared with sham group; ^#^ *p* < .05, ^##^ *p* < .01 compared with IRI with CIT of 0 h. Abbreviations: BUN, blood urea nitrogen; CIT, cold ischemic time; HTK, histidine‐tryptophan‐ketoglutarate; HPF, high‐power field; IRI, ischemia‐reperfusion injury; KT, kidney transplantation; MT, Masson's trichrome; PAS, periodic acid–Schiff; PBS, phosphate‐buffered saline; SEM, standard error of the mean; WIT, warm ischemic time.

Cold IRI was induced using various durations of CIT in syngeneic mouse models of kidney transplantation (Figure 1A). Mouse mortality occurred when the CIT was longer than 6 h and increased with increasing CIT (CIT 6 h vs. 7 h, *p* = .031; CIT 6 h vs. 8 h, *p* < .01; Figure 1B). Renal functions on day 1 were worse in the 6 h‐CIT group than those in the 0 h‐CIT and sham groups (Figure 1B). Renal tubular injury on day 1 was also more severe in the 6 h‐CIT group than in the 0 h‐CIT group (Figure 1C,D). Moreover, the renal cortical thickness was thinner and renal fibrosis was more severe in the 6 h‐CIT group than in the 0 h‐CIT group on day 28 (Figure 1E). The number of renal inflammatory cells (leukocytes, macrophages and T cells; Figure S3A) was higher in the CIT 6 h group than in the sham group or CIT 0 h group. In contrast, those of renal Tregs did not differ (Figure S3B). The CIT 6 h group had higher renal expression of fibrosis‐associated molecules (transforming growth factor beta [TGF‐β], alpha‐smooth muscle actin [α‐SMA], fibronectin and Collagen‐IV; Figure S3C) and α‐SMA expression in renal F4/80^+^CD11b^+^ macrophages (Figure S3D). Overall, cold IRI with a longer CIT induced acute renal injury and chronic fibrosis to a greater extent than that induced by the IRI with a CIT of 0 h.

### 3.2 Role of Tregs in renal cold IRI after kidney transplantation

**FIGURE 2:**
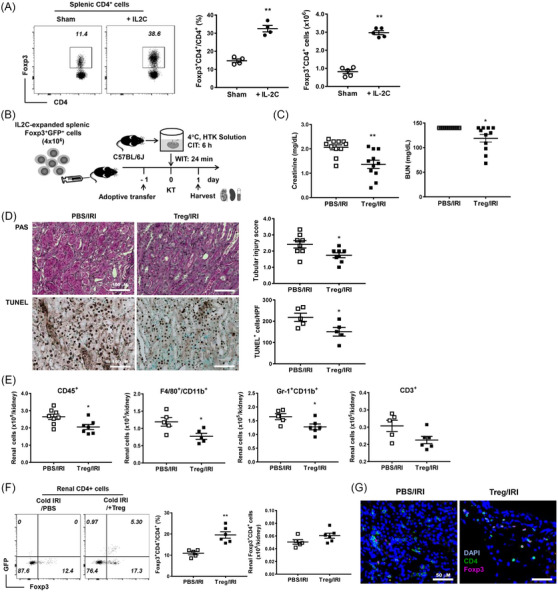
Adoptive transfer of IL‐2C‐treated Tregs attenuated renal cold IRI. (A) IL‐2C administration induced the expansion of CD4^+^Foxp3^+^ Tregs. (B) CD4^+^Foxp3^+^ Tregs were adoptively transferred to recipient mice 1 day before inducing cold IRI and kidneys were harvested along with blood sampling on day 1. (C) Level of blood creatinine and BUN. (D) Renal tissue injury scores and renal tubular apoptosis (based on TUNEL staining). Magnification, 200×. (E) Absolute numbers of renal CD45^+^, F4/80^+^CD11b^+^, Gr‐1^+^CD11b^+^ and CD3^+^ cells. (F) Proportions and absolute number of renal CD4^+^Foxp3^+^ Tregs. (G) Immunofluorescence images showing renal infiltration of CD4^+^Foxp3^+^ Tregs. Green, pink and blue colours indicate CD4, Foxp3 and DAPI, respectively. Magnification, 400×. Lines and whiskers in dot plots indicate the mean and SEM, respectively. *p < .05, **p < .01 compared with sham group (A) or PBS/IRI group (C–F). Abbreviations: BUN, blood urea nitrogen; CIT, cold ischemic time; DAPI, 4′,6‐diamidino‐2‐phenylindole; Foxp3, forkhead box P3; GFP, green fluorescent protein; HPF, high‐power field; HTK, histidine‐tryptophan‐ketoglutarate; IL‐2C, interleukin‐2/anti‐IL‐2 antibody immune complex; IRI, ischemia‐reperfusion injury; KT, kidney transplantation; PAS, periodic acid–Schiff; PBS, phosphate‐buffered saline; SEM, standard error of the mean; Tregs, regulatory T cells; TUNEL, terminal deoxynucleotidyl transferase dUTP nick‐end labelling; WIT, warm ischemic time.

IL‐2C therapy significantly increased the number of CD4^+^Foxp3^+^ Tregs in the spleen (Figure 2A). Subsequently, we sorted and adoptively transferred IL‐2C‐treated Tregs one day before kidney transplantation and assessed the renal outcomes on day 1 after cold IRI with a CIT of 6 h (Figure 2B). Treg transfer attenuated renal functional deterioration (Figure 2C). Furthermore, both renal tubular injury and apoptosis were attenuated via Treg therapy (Figure 2D). Treg transfer reduced the infiltration of CD45^+^ leukocytes, F4/80^+^CD11b^+^ macrophages and Gr‐1^+^CD11b^+^ neutrophils into kidneys and the renal T‐cell infiltration showed a decreasing trend in the Treg‐transfer group (Figure 2E). The Treg‐transfer group exhibited an increase in the percentage of renal Foxp3^+^CD4^+^ Tregs (Figure 2F,G). In‐vitro suppression assays also showed the sorted CD4^+^Foxp3^+^ Tregs successfully suppressed the proliferation of effector T cells in a dose‐dependent manner (Figure S4).

When splenic CD4^+^Foxp3^+^ Tregs from IL‐2C‐untreated mice were transferred one day before kidney transplantation (Figure S5A), the transfer of natural Tregs improved renal functions (Figure S5B) and attenuated renal tissue injury after cold IRI (Figure S5C). The natural Tregs also suppressed renal infiltration of inflammatory cells (Figure S5D). However, there was no significant increase in renal infiltration of Tregs in the natural Treg group (Figure S5E).

**FIGURE 3:**
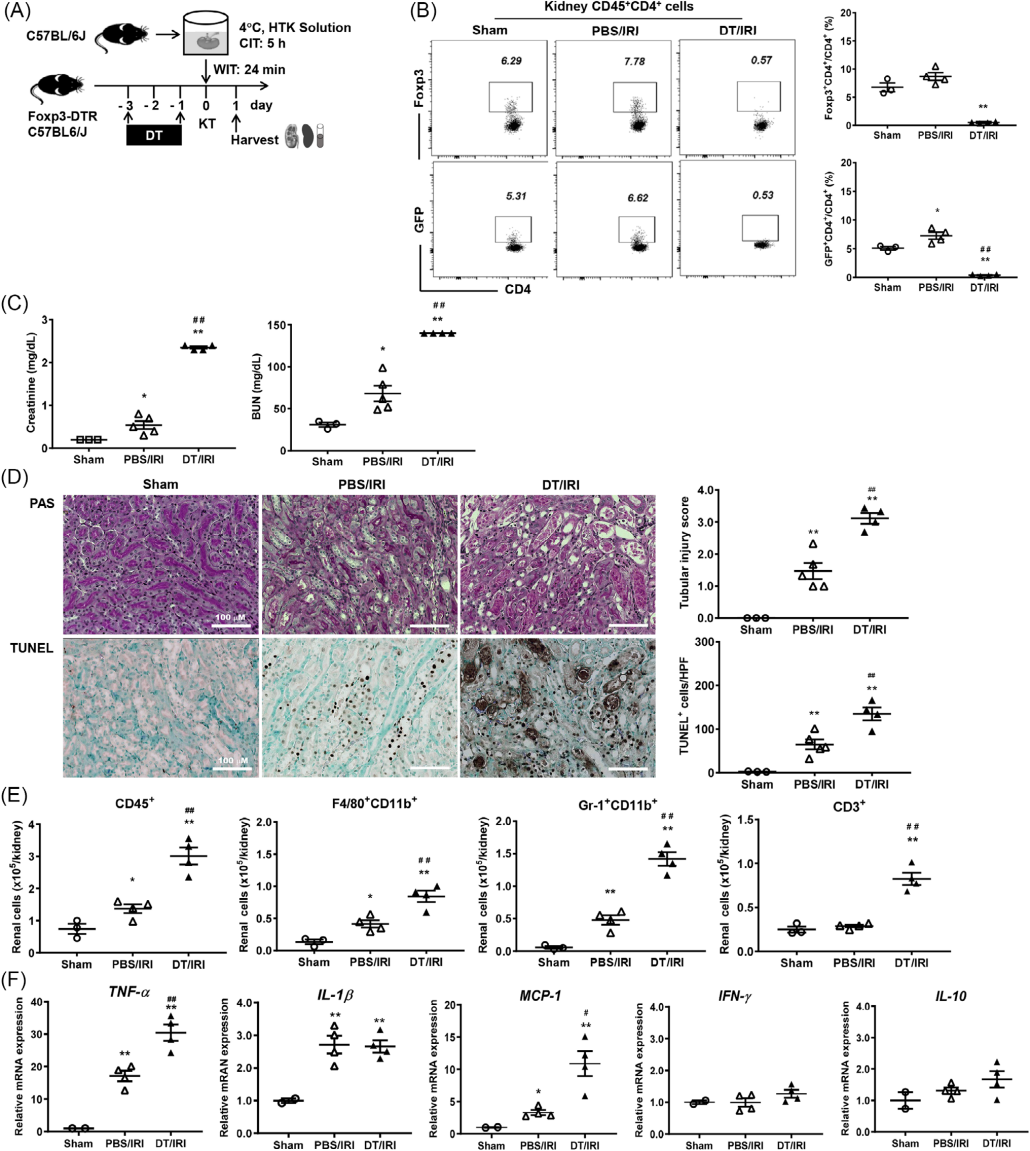
Treg depletion aggravated renal cold IRI after kidney transplantation. (A) Renal cold IRI (CIT 5 h) was induced with or without DT. Kidneys and the spleens along with blood samples were harvested on day 1 after cold IRI. (B) DT treatment depleted most of renal CD4^+^Foxp3^+^ Tregs. (C) Blood levels of creatinine and BUN. (D) Renal tissue injury scores and renal tubular apoptosis (based on TUNEL staining). Magnification, 200×. (E) Absolute numbers of renal CD45^+^, F4/80^+^CD11b^+^, Gr‐1^+^CD11b^+^ and CD3^+^ cells. (F) Renal mRNA expression level of *Tnfa*, *Il1b*, *Mcp1*, *Ifng* and *Il10* normalized to *Gapdh* expression level. Lines and whiskers in dot plots indicate the mean and SEM. *p < .05, **p < .01 compared to sham group. ^#^ *p* < .05, ^##^ *p* < .01 for DT/IRI group versus PBS/IRI group. Abbreviations: BUN, blood urea nitrogen; CIT, cold ischemic time; DT, diphtheria toxin; Foxp3, forkhead box P3; HTK, Histidine‐tryptophan‐ketoglutarate; HPF, high power field; *Gapdh*, glyceraldehyde 3‐phosphate dehydrogenase; IRI, ischemia‐reperfusion injury; IL, interleukin; *Ifng*, interferon‐γ; KT, Kidney transplantation; *Mcp1*, monocyte chemoattractant protein‐1; PAS, periodic acid–Schiff; PBS, phosphate‐buffered saline; SEM, standard error of the mean; *Tnfa*, tumour necrosis factor‐α; TUNEL, terminal deoxynucleotidyl transferase dUTP nick‐end labelling; WIT, warm ischemic time.

Next, we depleted renal Tregs via DT administration to Foxp3‐GFP‐DTR mice before inducing cold IRI with a CIT of 5 h (Figure 3A,B). The levels of creatinine and BUN were higher in the DT/IRI group compared to the phosphate‐buffered saline (PBS)/IRI group (Figure 3C). Furthermore, Treg depletion enhanced renal tissue damage and apoptosis (Figure 3D). Infiltration of CD45^+^, F4/80^+^CD11b^+^, Gr‐1^+^CD11b^+^ and CD3^+^ cells was also increased after Treg depletion (Figure 3E). Treg depletion further increased renal mRNA expression of tumour necrosis factor‐α (TNF‐α) and monocyte chemoattractant protein 1 (MCP‐1), which were induced after cold IRI (Figure 3F). Taken together, the Treg transfer attenuated renal cold IRI, while Treg depletion exacerbated cold IRI.

### 3.3 IL‐2C treatment mitigated acute renal damage in cold IRI

**FIGURE 4:**
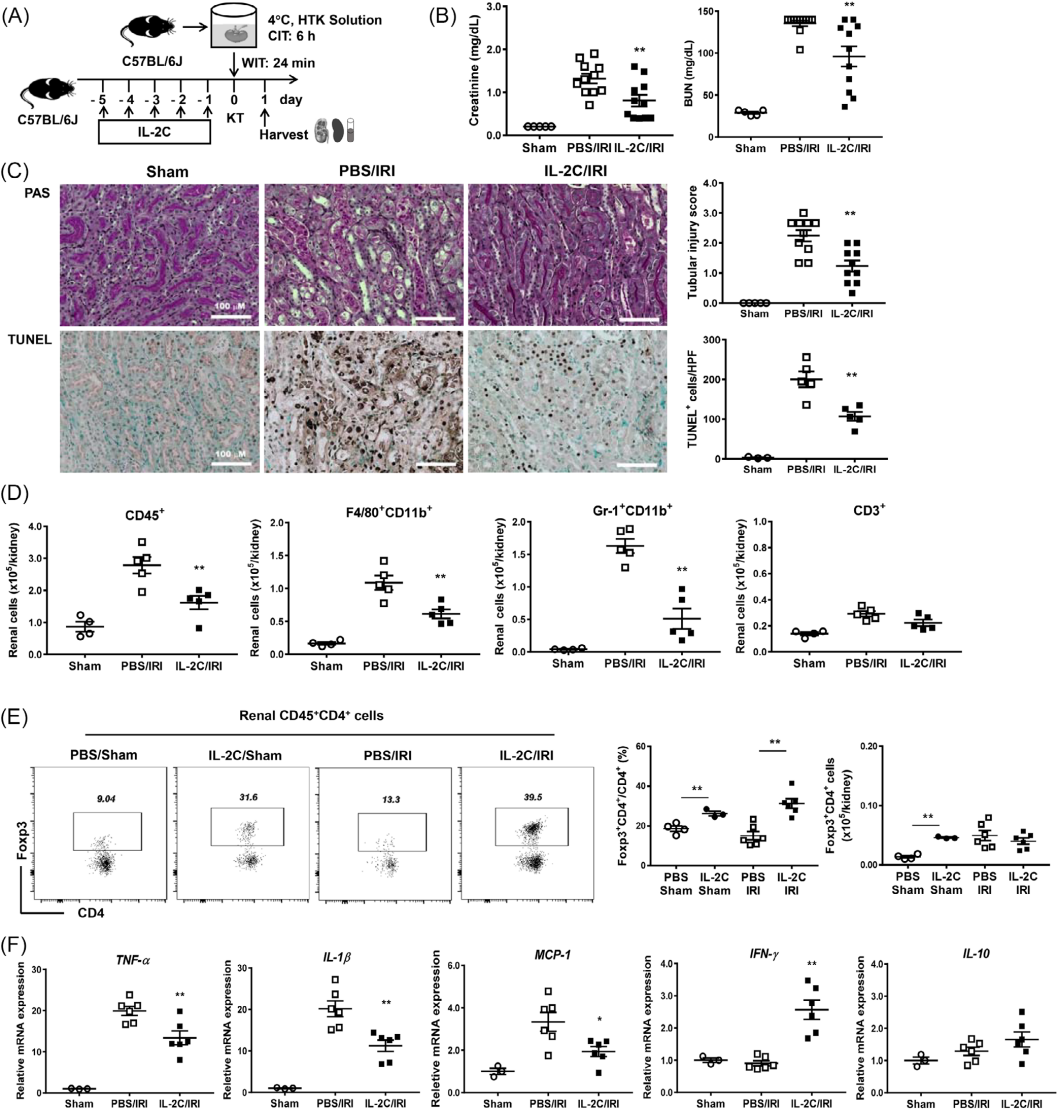
IL‐2C treatment attenuated acute renal injury in cold IRI. (A) IL‐2C or PBS was administered to recipient mice prior to inducing cold IRI. Kidneys and spleens along with blood samples were harvested on day 1 after inducing cold IRI. (B) The blood level of creatinine and BUN. (C) Renal tissue injury scores and renal tubular apoptosis (based on TUNEL staining). Magnification, 200×. (D) Absolute number of renal CD45^+^, F4/80^+^CD11b^+^, Gr‐1^+^CD11b^+^ and CD3^+^ cells. (E) Flow cytometric diagrams for renal CD4^+^Foxp3^+^ Tregs with proportions and absolute number of renal CD4^+^Foxp3^+^ Tregs. (F) Renal mRNA expression level of *Tnfa*, *Il1b*, *Mcp1*, *Ifng* and *Il10* normalized to *Gapdh* expression level. Lines and whiskers in dot plots indicate the mean and SEM. *p < .05, **p < .01 for PBS group versus IL‐2C group. Abbreviations: BUN, blood urea nitrogen; CIT, cold ischemic time; Foxp3, forkhead box P3; *Gapdh*, glyceraldehyde 3‐phosphate dehydrogenase; HTK, histidine‐tryptophan‐ketoglutarate; HPF, high‐power field; *Ifng*, interferon‐γ; IL, interleukin; IL‐2C, IL‐2/anti‐IL‐2 antibody immune complex; IRI, ischemia‐reperfusion injury; KT, kidney transplantation; *Mcp1*, monocyte chemoattractant protein‐1; PAS, periodic acid–Schiff; PBS, phosphate‐buffered saline; SEM, standard error of the mean; *Tnfa*, tumour necrosis factor‐α; TUNEL, terminal deoxynucleotidyl transferase dUTP nick‐end labelling; WIT, warm ischemic time.

IL‐2C was daily administered from day −5 to day −1 before kidney transplantation and the renal outcomes were assessed on day 1 after inducing cold IRI (Figure 4A). IL‐2C treatment led to a significant reduction in blood creatinine and BUN levels compared to the control group treated with PBS (Figure 4B). Additionally, IL‐2C therapy reduced the tissue injury scores and apoptosis of renal tubular epithelial cells (Figure 4C). It also reduced renal infiltration of CD45^+^ leukocytes, including macrophages and neutrophils (Figure 4D and Figure S6). IL‐2C therapy increased the proportions of Tregs in kidneys (Figure 4E and Figure S7A) and spleens (Figure S7B) in comparison to the PBS control, confirming IL‐2C‐induced Treg expansion. In addition, IL‐2C therapy reduced renal infiltration of NK1.1^+^CD3^−^ NK cells (Figure S8). Although IL‐2C did not induce change in the number of total innate lymphoid cells (ILCs, CD45^+^Lin^−^CD127^+^) in kidney tissues, it increased renal infiltration of regulatory ILCs (ILCregs, CD45^+^Lin^−^CD127^+^CD90^+^IL‐10^+^) (Figure S8). Renal mRNA expression levels of TNF‐α (*Tnfa*), IL‐1β (*Il1b*) and MCP‐1 (*Mcp1*) in the IL‐2C group were lower than those in the PBS group (Figure 4F). Notably, the renal mRNA expression level of interferon‐γ (IFN‐γ, *Ifng*) in the IL‐2C group was higher than that in the PBS group, and IL‐10 (*Il10*) expression was increased in the IL‐2C group, although the change was not statistically significant (Figure 4F). Overall, IL‐2C therapy improved kidney function and mitigated tissue damage and renal inflammation in the acute phase of cold IRI.

### 3.4 IL‐2C treatment mitigated subacute renal injury and promoted renal recovery in cold IRI

**FIGURE 5:**
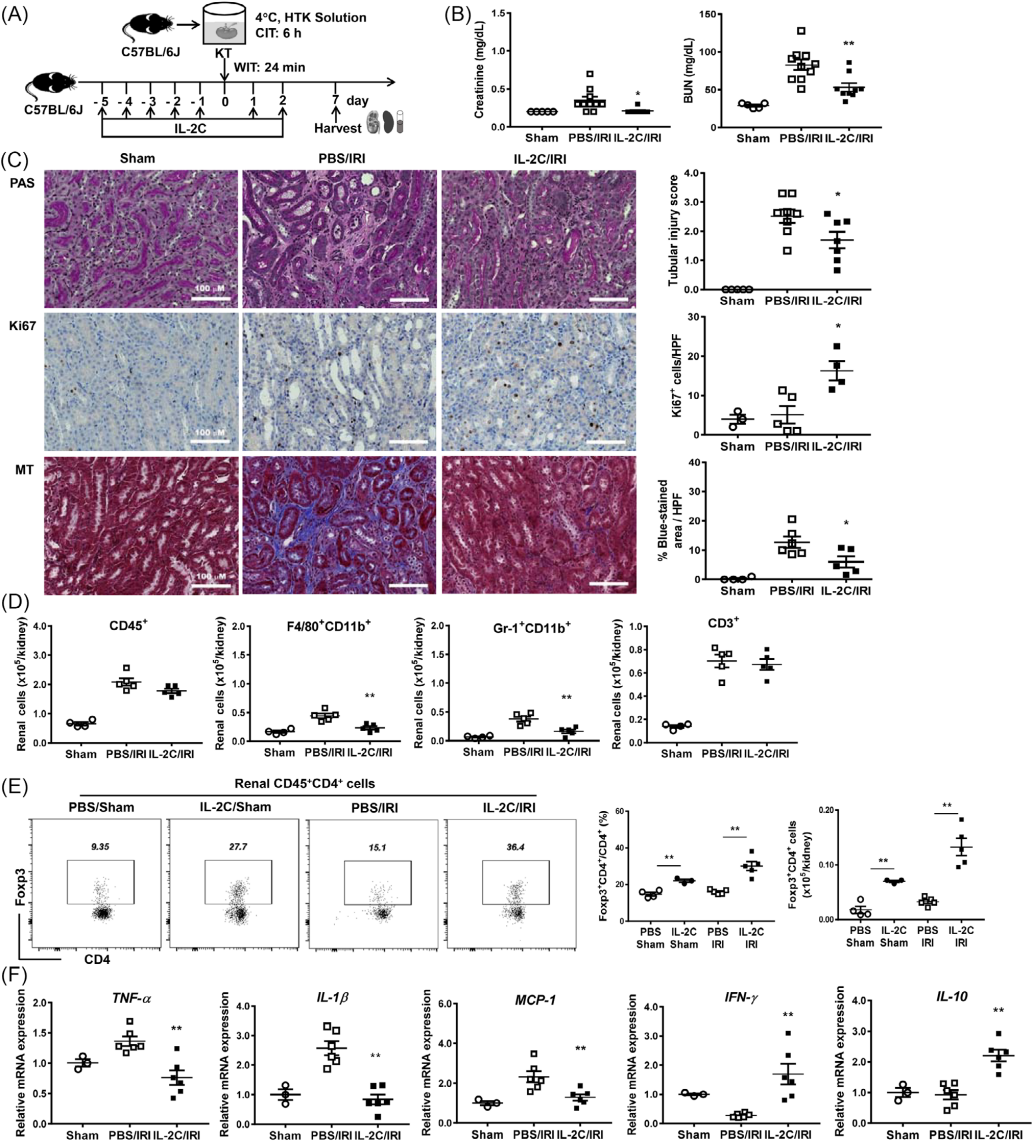
IL‐2C treatment attenuated subacute renal injury and facilitated renal recovery in cold IRI. (A) IL‐2C or PBS was administered to recipient mice seven times up to day 2 after cold IRI. Kidneys and spleens along with blood samples were harvested on day 7 after cold IRI. (B) The blood level of creatinine and BUN. (C) Renal tissue injury scores (PAS staining), renal tubular regeneration (Ki67 staining) and renal fibrosis (MT staining). Magnification, 200×. (D) Absolute numbers of renal CD45^+^, F4/80^+^CD11b^+^, Gr‐1^+^CD11b^+^ and CD3^+^ cells. (E) Flow cytometric diagrams for renal CD4^+^Foxp3^+^ Tregs with proportions and absolute numbers of renal CD4^+^Foxp3^+^ Tregs. (F) Renal mRNA expression levels of *Tnfa*, *Il1b*, *Mcp1*, *Ifng* and *Il10* normalized to *Gapdh* expression level. Lines and whiskers in dot plots indicate the mean and SEM. *p < .05, **p < .01 for IL‐2C group versus PBS group. Abbreviations: BUN, blood urea nitrogen; CIT, cold ischemic time; Foxp3, forkhead box P3; *Gapdh*, glyceraldehyde 3‐phosphate dehydrogenase; HTK, histidine‐tryptophan‐ketoglutarate; HPF, high‐power field; *Ifng*, interferon‐γ; IL, interleukin; IL‐2C, IL‐2/anti‐IL‐2 antibody immune complex; IRI, ischemia‐reperfusion injury; KT, kidney transplantation; *Mcp1*, monocyte chemoattractant protein‐1; MT, Masson's trichrome; PAS, periodic acid–Schiff; PBS, phosphate‐buffered saline; SEM, standard error of the mean; *Tnfa*, tumour necrosis factor‐α; WIT, warm ischemic time.

IL‐2C was daily administered from the day −5 to −1 before transplantation and from day 1 to 2 after transplantation. We assessed the effects of IL‐2C treatment on the subacute period of cold IRI on day 7 (Figure 5A). IL‐2C treatment improved renal functions (Figure 5B). Moreover, IL‐2C treatment reduced tissue damage promoted renal regeneration (Ki67) and attenuated renal fibrosis (Figure 5C). Furthermore, renal tubular expression of AQP‐1 and VEGF, markers of renal differentiation and regeneration, was also increased by IL‐2C (Figure S9).^30^, ^31^, ^32^ Renal infiltration of macrophages and neutrophils was suppressed following IL‐2C treatment (Figure 5D). IL‐2C therapy increased the Treg's number in kidneys and spleens compared with the PBS control (Figure 5E and Figure S7B). The renal expression levels of *Tnfa*, *Il1b* and *Mcp1* were significantly lower and those of *Ifng* and *Il10* were significantly higher in the IL‐2C group than those in the control group (Figure 5F). Therefore, IL‐2C treatment mitigated subacute renal damage and promoted renal recovery following cold IRI.

### 3.5 IL‐2C therapy attenuated chronic renal fibrosis after cold IRI

**FIGURE 6:**
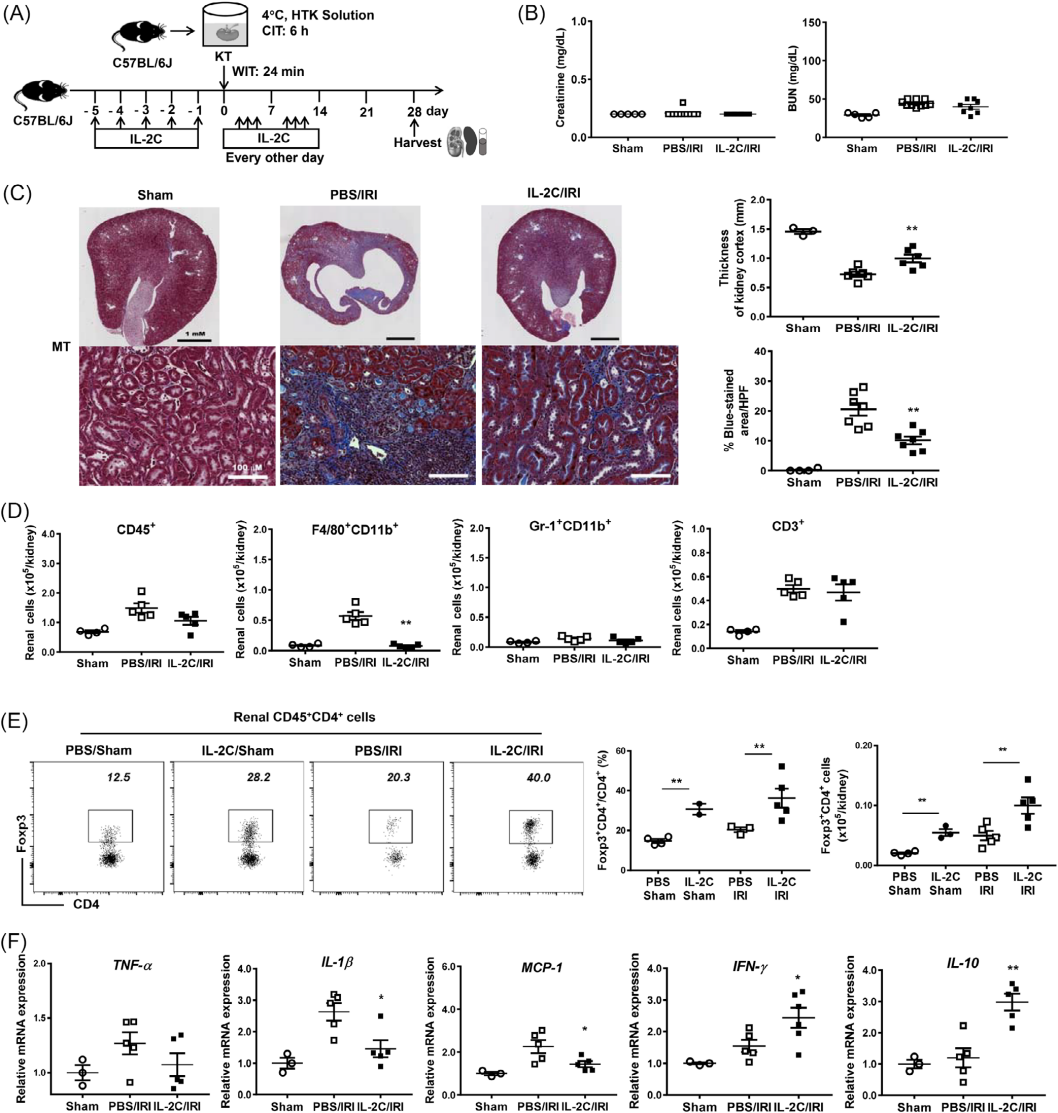
IL‐2C treatment protected kidneys against cold IRI in the chronic phase. (A) IL‐2C or PBS was administered to recipient mice for five consecutive days before inducing cold IRI and thrice a week up to 2 weeks after cold IRI. Kidneys and spleens along with blood samples were harvested on day 28. (B) The blood level of creatinine and BUN. (C) Renal cortical thickness (magnification, 40×; upper panels) and fibrosis (magnification, 200×; lower panels). (D) Absolute numbers of renal CD45^+^, F4/80^+^CD11b^+^, Gr‐1^+^CD11b^+^ and CD3^+^ cells. (E) Flow cytometric diagrams for renal CD4^+^Foxp3^+^ Tregs with proportions and absolute numbers of renal CD4^+^Foxp3^+^ Tregs. (F) Renal mRNA expression level of *Tnfa*, *Il1b*, *Mcp1*, *Ifng* and *Il10* normalized to *Gapdh* expression level. Lines and whiskers in dot plots indicate the mean and SEM, respectively. *p < .05, **p < .01 for IL‐2C group versus PBS group. Abbreviations: BUN, blood urea nitrogen; CIT, cold ischemic time; Foxp3, forkhead box P3; GAPDH, glyceraldehyde 3‐phosphate dehydrogenase; HTK, histidine‐tryptophan‐ketoglutarate; HPF, high‐power field; *Ifng*, interferon‐γ; IL, interleukin; IL‐2C, IL‐2/anti‐IL‐2 antibody immune complex; IRI, ischemia‐reperfusion injury; KT, kidney transplantation; *Mcp1*, monocyte chemoattractant protein‐1; MT, Masson's trichrome; PBS, phosphate‐buffered saline; SEM, standard error of the mean; *Tnfa*, tumour necrosis factor‐α; WIT: warm ischemic time.

IL‐2C was daily administered from the day −5 to −1 before transplantation, thrice a week for the first two weeks after transplantation, and the renal outcomes were assessed on day 28 (Figure 6A). Renal functions were similar among different groups at this time point (Figure 6B). However, the IL‐2C group exhibited higher renal cortical thickness and lower renal fibrosis than the control group (Figure 6C). IL‐2C treatment decreased the number of renal F4/80^+^CD11b^+^ macrophages (Figure 6D) and increased the renal Treg's number (Figure 6E). Moreover, the renal mRNA expression levels of *Il1b* and *Mcp1* were lower and those of *Ifng* and *Il10* were higher in the IL‐2C group than those in the control group (Figure 6F).

**FIGURE 7:**
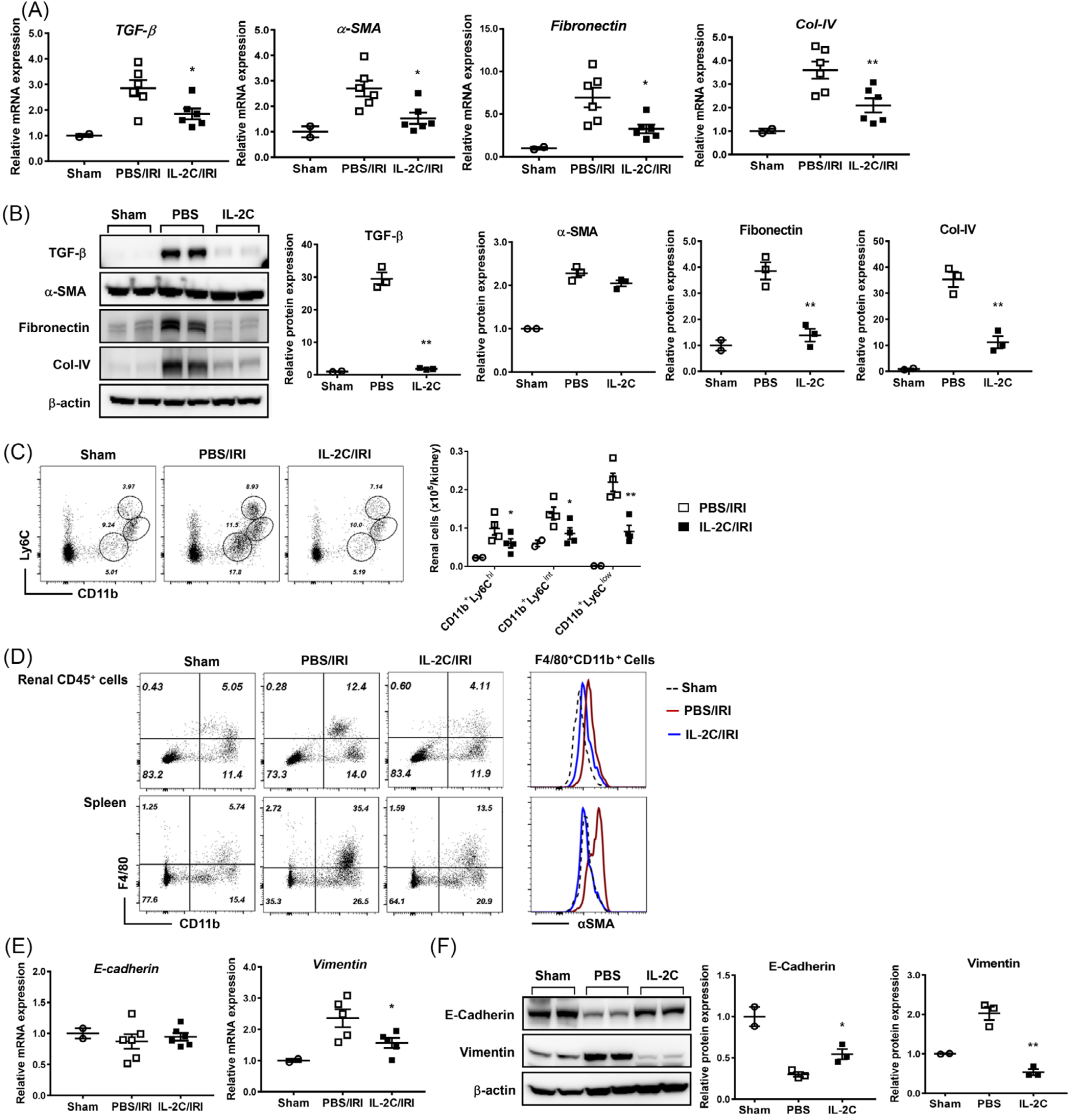
IL‐2C treatment attenuated chronic renal fibrosis after cold IRI. (A) Renal mRNA expression level of *TGF‐β* (*Tgfb1)*, *α‐SMA* (*Acta2*), *fibronectin (Fn1)* and *Col‐IV (Col4a1)* normalized to *Gapdh* expression level on day 28 after cold IRI. (B) Renal deposition of TGF‐β, αSMA, fibronectin and Col‐IV was measured via western blotting and normalized to β‐actin levels. (C) Absolute numbers of renal CD11b^+^Ly6C^high^, CD11b^+^Ly6C^int^ and CD11b^+^Ly6C^low^ cells. (D) Expression of α‐SMA in renal and splenic F4/80^+^CD11b^+^ macrophages. (E) mRNA expression levels of renal *E‐cadherin* (*Cdh1*) and *vimentin (Vim)* normalized to *Gapdh* expression level. (F) Renal deposition of E‐cadherin and vimentin was measured via western blotting and normalized to β‐actin levels. Lines and whiskers in dot plots indicate the mean and SEM, respectively. *p < .05, **p < .01 for IL‐2C/IRI group versus PBS/IRI group. Abbreviations: αSMA, α‐smooth muscle actin; Col‐IV, type IV collagen; E‐cadherin, epithelial cadherin; *Gapdh*, glyceraldehyde 3‐phosphate dehydrogenase; IL‐2C, IL‐2/anti‐IL‐2 antibody immune complex; IRI, ischemia‐reperfusion injury; PBS, phosphate‐buffered saline; SEM, standard error of the mean; TGF‐β, transforming growth factor‐β.

When renal expression levels of fibrosis‐associated molecules were assessed on day 28, the mRNA and protein expression levels of TGF‐β, α‐SMA, fibronectin and type IV collagen were reduced by IL‐2C therapy (Figure 7A,B and Figure S2A). The renal infiltration of profibrotic CD11b^+^Ly6C^low^ macrophages was mitigated by IL‐2C therapy (Figure 7C). The α‐SMA expression levels in renal and splenic F4/80+CD11b+ macrophages were lower in the IL‐2C group than in the PBS group (Figure 7D). When the impact of IL‐2C on epithelial‐to‐mesenchymal transition in cold IRI was assessed, IL‐2C treatment was found to increase E‐cadherin expression and decrease vimentin expression at mRNA (Figure 7E) or protein (Figure 7F and Figure S2B) levels. Furthermore, immunohistochemical staining revealed that IL‐2C therapy suppressed renal α‐SMA expression and increased renal E‐cadherin expression on day 28 after IRI (Figure S10). Overall, IL‐2C therapy reduced the severity of chronic renal fibrosis after cold IRI.

### 3.6 IL‐2C treatment suppressed ROS generation and enhanced antioxidant function in cold IRI

We assessed the impact of IL‐2C treatment on the production of ROS on day 7 and 28. IL‐2 treatment suppressed the level of 8‐OHdG and MDA in both plasma and kidneys while it increased plasma and renal GSH levels and SOD activity (Figure S11A). Additionally, renal expression of nicotinamide adenine dinucleotide phosphate oxidase (Nox2) on days 7 and 28 was suppressed by IL‐2C therapy (Figure S11B,C and Figure S11C). Furthermore, DHE staining revealed that IL‐2C therapy mitigated the rise in the ROS content of kidney tissues following IRI (Figure S11D). Overall, IL‐2C treatment suppressed ROS generation and enhanced antioxidant function in renal cold IRI.

### 3.7 IL‐2C treatment suppressed systemic inflammation after renal IRI

We measured systemic levels of proinflammatory cytokines on days 1 and 28 after renal IRI. Systemic concentrations of IL‐1β, TNF‐α, IL‐6 and IFN‐γ on days 1 and 28 were suppressed in the IL‐2C group, supporting the safety of IL‐2C therapy (Figure S12).

### 3.8 IL‐2C therapy after renal IRI promoted renal recovery and inhibited renal fibrosis in cold IRI

**FIGURE 8:**
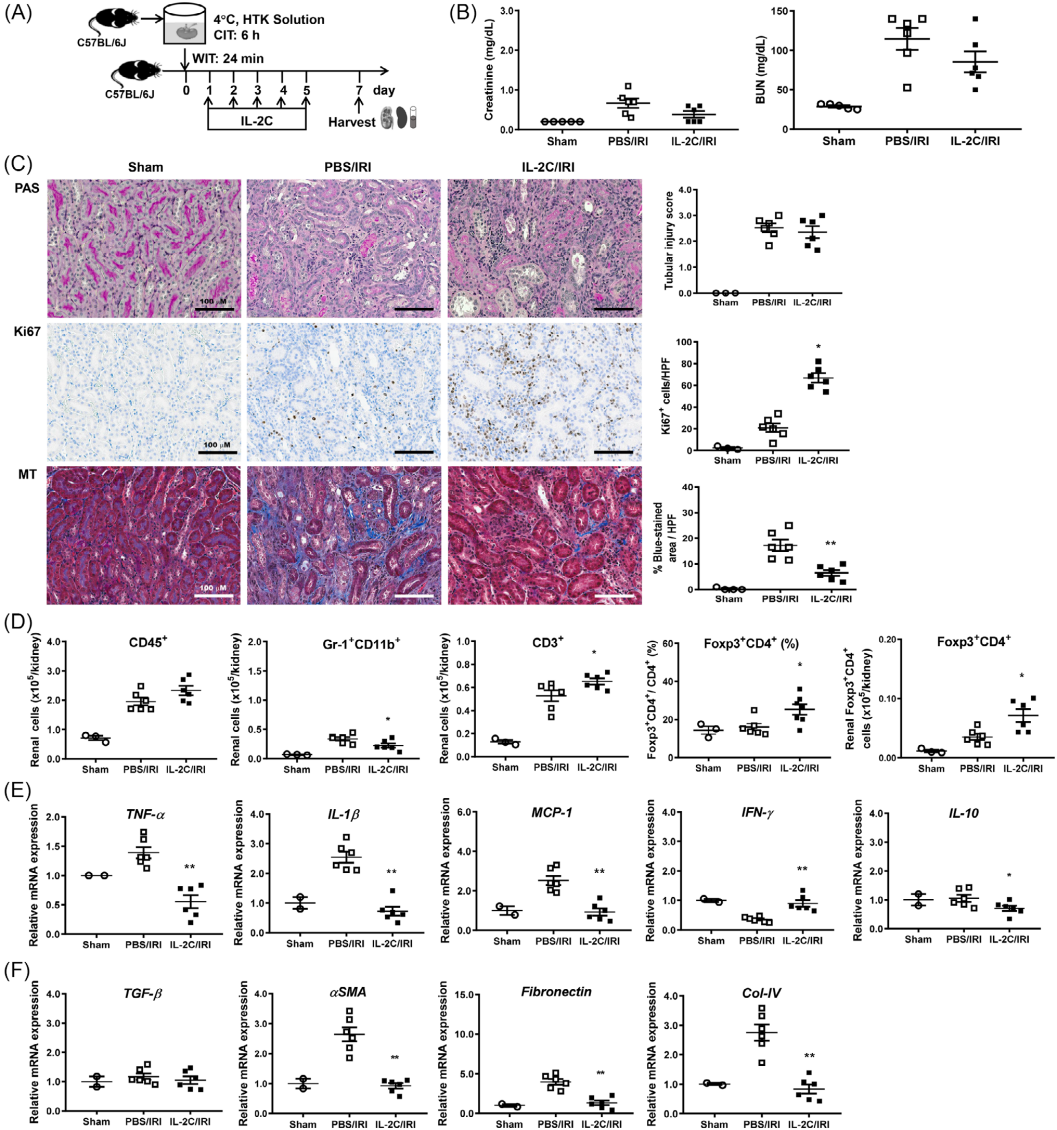
IL‐2C treatment after IRI facilitated renal recovery and suppressed renal fibrosis in cold IRI. (A) IL‐2C or PBS was administered to recipient mice five times from day 1–5 after cold IRI. Kidneys and spleens along with blood samples were harvested on day 7 after cold IRI. (B) The blood level of creatinine and BUN. (C) Renal tissue injury scores (PAS staining), renal tubular regeneration (Ki67 staining) and renal fibrosis (MT staining). Magnification, 200×. (D) Absolute numbers of renal CD45^+^, Gr‐1^+^CD11b^+^ and CD3^+^ cells. Proportions and absolute numbers of renal CD4^+^Foxp3^+^ Tregs. (E) Renal mRNA expression levels of *Tnfa*, *Il1b*, *Mcp1*, *Ifng* and *Il10* normalized to *Gapdh* expression level. (F) Renal mRNA expression level of *TGF‐β* (*Tgfb1)*, *α‐SMA* (*Acta2*), *fibronectin* (*Fn1*) and *Col‐IV* (*Col4a1*) normalized to *Gapdh*. Lines and whiskers in dot plots indicate the mean and SEM, respectively. *p < .05, **p < .01 for IL‐2C/IRI group versus PBS/IRI group. Abbreviations: αSMA, α‐smooth muscle actin; BUN, blood urea nitrogen; CIT, cold ischemic time; Col‐IV, type IV collagen; Foxp3, forkhead box P3; *Gapdh*, glyceraldehyde 3‐phosphate dehydrogenase; HTK, histidine‐tryptophan‐ketoglutarate; HPF, high‐power field; *Ifng*, interferon‐γ; IL‐2C, IL‐2/anti‐IL‐2 antibody immune complex; IRI, ischemia‐reperfusion injury; *Mcp1*, monocyte chemoattractant protein‐1; MT, Masson's trichrome; PAS, periodic acid–Schiff; PBS, phosphate‐buffered saline; SEM, standard error of the mean; TGF‐β, transforming growth factor‐β; *Tnfa*, tumor necrosis factor‐α; WIT, warm ischemic time.

IL‐2C was daily administered from days 1–5 and the impact of post‐IRI IL‐2C treatment on the subacute phase of cold IRI was assessed on day 7 (Figure 8A). IL‐2C treatment did not significantly improve renal functions or tissue injury scores (Figure 8B,C). However, IL‐2C treatment facilitated renal regeneration and attenuated renal fibrosis (Figure 8C). In parallel with results in pre‐AKI treatment models, post‐AKI IL‐2C treatment also increased both the proportion and number of renal Tregs compared to PBS treatment (Figure 8D). The renal expression levels of *Tnfa*, *Il1b* and *Mcp1* were lower and those of *Ifng* were higher in the IL‐2C group than those in the control group (Figure 8E). The mRNA levels of *αSMA*, *fibronectin* and *Col‐IV* were decreased following IL‐2C therapy (Figure 8F). Thus, post‐AKI IL‐2C treatment improved kidney recovery and inhibited kidney fibrosis in cold IRI similar to pre‐AKI IL‐2C treatment.

### 3.9 Treg depletion eliminated the protective impact of IL‐2C on renal cold IRI

**FIGURE 9:**
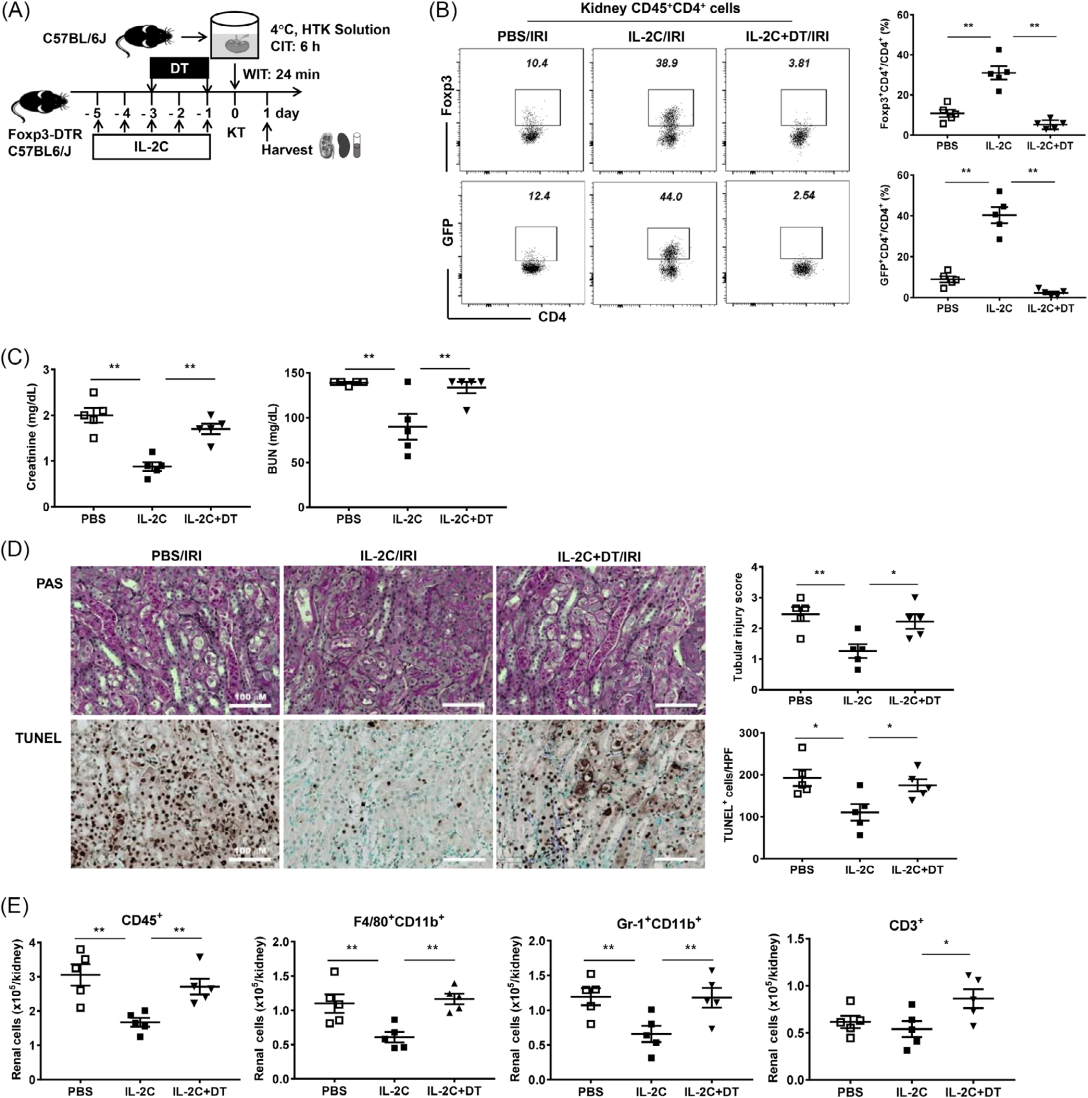
Treg depletion abrogated the beneficial effects of IL‐2C on renal cold IRI. (A) IL‐2C was administered to recipient mice prior to inducing cold IRI with or without DT. Kidneys and spleens along with blood samples were harvested on day 1 after inducing cold IRI. (B) DT treatment nearly depleted renal CD4^+^Foxp3^+^ Tregs. (C) The blood level of creatinine and BUN. (D) Renal tissue injury scores and renal tubular apoptosis (based on TUNEL staining). Magnification, 200×. (E) Absolute numbers of renal CD45^+^, F4/80^+^CD11b^+^, Gr‐1^+^CD11b^+^ and CD3^+^ cells. Lines and whiskers in dot plots indicate the mean and SEM, respectively. *p < .05, **p < .01. Abbreviations: BUN, blood urea nitrogen; CIT, cold ischemic time; DT, diphtheria toxin; Foxp3, forkhead box P3; HTK, histidine‐tryptophan‐ketoglutarate; HPF, high‐power field; IL‐2C, IL‐2/anti‐IL‐2 antibody immune complex; IRI, ischemia‐reperfusion injury; KT, Kidney transplantation; PAS, periodic acid–Schiff; PBS, phosphate‐buffered saline; SEM, standard error of the mean; TUNEL, terminal deoxynucleotidyl transferase dUTP nick‐end labelling; WIT, warm ischemic time.

To confirm Tregs’ role in the IL‐2C‐mediated protective effects on cold IRI, we administered DT (day −3 and day −1) with IL‐2C (daily from day −5 to −1) to Foxp3‐GFP‐DTR mice before inducing cold IRI (Figure 9A) and found a reduction in the number of renal Tregs in the IL‐2C+DT group (Figure 9B). Along with Treg depletion, the improvement in renal function mediated by IL‐2C therapy was nearly abrogated in the IL‐2C+DT group (Figure 9C). Furthermore, Treg depletion eliminated the protective effects of IL‐2C on renal tissue damage and renal apoptosis (Figure 9D). The renal infiltration of macrophages, neutrophils and T cells increased in the IL‐2C+DT group compared to that in the IL‐2C group (Figure 9E).

## 4 DISCUSSION

This study revealed that cold IRI with a longer CIT induced more severe acute injury and chronic fibrosis than that induced by renal IRI with a CIT of 0 h in kidney transplantation. Tregs play a crucial role in protecting kidneys from cold IRI. Furthermore, IL‐2C treatment attenuated renal injury and subsequent fibrosis in the acute, subacute and chronic periods of cold IRI by increasing the renal Treg population.

Presently, mouse models of warm IRI have been used widely owing to the technical difficulties of developing a mouse model of cold IRI, and the results of warm IRI models are typically extrapolated to renal cold IRI associated with kidney transplantation. Recently, a mouse model of cold IRI has recently been developed successfully for studying cold IRI.^33^, ^34^, ^35^ The mouse survival rates after inducing cold IRI according to the CIT in the present study clearly support well‐established mouse cold IRI models.

Renal cold IRI includes an additional process of cold storage with cold ischemia, causing more severe injury than that caused by warm IRI. Renal cold IRI results in poor allograft outcomes, such as delayed graft function (DGF) and allograft rejection.^8^, ^36^ The increase in CIT is followed by more severe renal cold IRI.^8^ The adjusted odds ratios of DGF according to CIT have been reported to be 1.8 (CIT ≥ 1 h), 2.5 (CIT ≥ 5 h), 3.3 (CIT ≥ 10 h) and 4.4 (CIT ≥ 15 h).^37^ Moreover, prolonged CIT (> 36 h) is associated with decreased allograft survival despite full human leukocyte antigen‐matching conditions.^1^ We demonstrated that cold IRI with a CIT longer than 6 h was associated with mortality or worse renal functions when compared with those with shorter CIT. Furthermore, cold IRI with a CIT of 6 h resulted in more severe renal chronic fibrosis than that observed in renal IRI of a CIT of 0 h, supporting the importance of minimizing the CIT for better long‐term graft outcomes.

Until now, many preservation solutions, such as the University of Wisconsin Solution, HTK solution and Celsior solution, have been developed to attenuate renal cold IRI.^38^, ^39^ Hypothermic and normothermic machine perfusion techniques have recently been developed to suppress DGF and graft failure, particularly for donor kidneys of low quality, such as expanded criteria donor and donation after circulatory death.^8^, ^40^, ^41^ Machine perfusion allows a continuous supply of oxygen and nutrients to organs during the preservation period, limiting cold ischemia.^40^ However, the beneficial effects of preservation solutions and machine perfusion on cold IRI are not sufficient, and therefore, additional strategies are required to control cold IRI. In addition to controlling cold IRI on the donor side using preservation solutions or machine perfusion, cold IRI should be managed on the recipient side. As inflammatory processes represent the key response in the development of cold IRI, altering the immunological balance toward regulatory cells over effector immune cells may represent a good therapeutic approach for controlling renal cold and warm IRI.^7^ Role of Tregs in renal warm IRI without kidney transplantation have been confirmed.^10^, ^11^ We found that Treg transfer mitigated acute damage and suppressed kidney inflammation following cold IRI. Additionally, Treg depletion via DT treatment aggravated acute injury and renal inflammation after cold IRI. These data confirmed that Tregs have a significant impact on renal cold IRI.

Cell therapy including Treg therapy involves strict manufacturing processes, and it is difficult to prepare autologous Tregs for every patient. Therefore, in‐vivo Treg induction therapy may represent a more practical approach than Treg therapy itself. JES6‐1, one of the anti‐IL‐2 monoclonal antibodies binds to the IL‐2 receptor β/γ‐binding site of IL‐2 and therefore allows IL‐2/JES6‐1 complexes to bind to only cells expressing IL‐2 receptor α, CD25.^20^, ^42^ Moreover, IL‐2C show increased half‐life of IL‐2 by 20–40 times.^42^ Through these mechanisms, IL‐2C complexes can induce preferential expansion of Tregs with high expression levels of CD25 over effector T and NK cells that mainly express IL‐2 receptor β/γ. Another Treg‐inducing strategy is IL‐2 muteins engineered to have decreased affinity to IL‐2 receptor β and increased CD25 dependence.^43^ IL‐2 muteins without complexing with anti‐IL‐2 can induce preferential expansion of Tregs and control autoimmunity.^43^ Both strategies can be applied to induce preferential expansion of Tregs; however, we favour IL‐2C, because anti‐IL‐2 antibodies in IL‐2C protect IL‐2 from scavengers and prolong its half‐life to a greater extent than IL‐2 muteins.^42^

We previously demonstrated that IL‐2C therapy can ameliorate renal warm IRI by increasing the population of renal Tregs.^12^ Based on these previous findings, we tested whether IL‐2C therapy could attenuate renal cold IRI in the context of kidney transplantation. We found that IL‐2C therapy improved renal functions, attenuated tissue injury and renal apoptosis, and suppressed renal inflammation in the acute period of cold IRI. Furthermore, IL‐2C therapy improved renal functions, promoted renal regeneration and inhibited fibrosis in the subacute phase of renal cold IRI. More importantly, IL‐2C therapy administered after IRI promoted renal recovery and reduced fibrosis, similar to IL‐2C therapy before IRI, suggesting a potential role of convenient IL‐2C therapy in clinical application to control renal cold IRI.

Acute kidney injury (AKI) increases the risk of progressive chronic kidney disease (CKD).^44^ Maladaptive responses of tubular epithelial cells to AKI cause dedifferentiation, epithelial‐to‐mesenchymal transition, cell cycle arrest, chronic inflammation and fibrogenesis.^45^, ^46^ In this study, the AKI‐to‐CKD transition was more remarkable in cold IRI with a long CIT than in that with a short CIT. Our study demonstrated that IL‐2C treatment successfully attenuated renal inflammation and fibrosis along with epithelial‐to‐mesenchymal transition in the chronic phase of renal cold IRI. CD11b^+^Ly6C^low^ macrophages are reported to function as major profibrotic macrophages after renal IRI and promote IRI‐induced CKD progression.^47^, ^48^ We found that CD11b^+^Ly6C^low^ macrophages were dominant in the chronic phase after cold IRI, and IL‐2C therapy significantly suppressed their infiltration into the kidneys. α‐SMA expression in macrophages can reflect the macrophage‐to‐myofibroblast transition and fibrosis process.^49^, ^50^ α‐SMA expression in F4/80^+^CD11b^+^ macrophages was remarkably suppressed via IL‐2C therapy in the chronic period of cold IRI. These data support that treatment with IL‐2C reduced the infiltration of profibrotic macrophages into kidneys and prevented the subsequent chronic renal fibrosis.

IL‐2C treatment reduced Nox2 expression and ROS‐mediated injury, such as DNA damage (8‐OHdG), lipid peroxidation (MDA) and DHE, while improving antioxidant function (GSH and SOD), thereby attenuating cold IRI. The protective effects of Tregs on ROS‐mediated injury might be indirect through their suppressive effects against immune cells, although exact mechanisms are unclear. Notably, IL‐2C therapy increased the renal expression level of IFN‐γ and IL‐10 and decreased those of TNF‐α, IL‐1β and MCP‐1 in cold IRI. IFN‐γ has been considered to either exert anti‐inflammatory or pro‐inflammatory effects according to the context.^51^ The upregulation of IFN‐γ observed in this study might have been induced by activated Tregs and contributed to their suppressive function.^52^

IL‐2C therapy remarkably increased the renal Treg's number or proportion in the acute, subacute and chronic phases of renal cold IRI.^12^ IL‐2C treatment also increased renal ILCregs. Because ILCregs showed renoprotective effects against warm IRI in recombinase recombination activation gene (RAG)‐knockout mice, where adaptive Tregs are absent,^19^ we cannot exclude a potential contribution of ILCregs to beneficial roles of IL‐2C in renal cold IRI. However, the number of renal ILCregs was much less than that of renal Tregs. Furthermore, the depletion of increased renal Tregs via DT treatment despite IL‐2C therapy reversed the protective effects of IL‐2C from IRI, suggesting that the major mechanism of IL‐2C‐mediated renal protection against cold IRI is the increase in renal Treg population.

The beneficial effects of IL‐2C on cold IRI could be influenced by the concomitant use of immunosuppressants in clinical transplantation. For example, basiliximab is a chimeric anti‐IL‐2 receptor alpha (CD25) monoclonal antibody, which binds to CD25 and thereby might weaken the IL‐2C‐mediated IL‐2 receptor signalling in Tregs, although basiliximab did not interfere with suppressive functions of Tregs.^53^ However, basiliximab treatment also binds to CD25 in recently activated effector T cells and can compromise IL‐2C‐mediated stimulation of activated effector T cells similarly to that of Tregs. Because the balance between effector T cells and Tregs is important for IRI as well as acute rejection, potential interference of basiliximab with IL‐2C‐mediated Treg expansion might be compensated by simultaneous interference of basiliximab with IL‐2C‐mediated expansion of activated effector T cells. Moreover, CD25^+^ Tregs were not depleted and still detected in the patients treated with basiliximab,^54^ suggesting that basiliximab‐mediated Treg decrease is not complete and remaining Tregs could expand in response to IL‐2C treatment. Further studies to elucidate the influence of concomitant immunosuppressants on IL‐2C‐mediated renoprotection against IRI are needed for future clinical application of IL‐2C in transplant patients.

This study had a few limitations. First, mouse models of cold IRI have certain disadvantages, such as inbred strains with low innate immune stimuli and different kidney anatomies from those of humans.^36^ Therefore, the present results should be verified in large animals, including nonhuman primates. Second, we utilized a syngeneic, cold IRI model to specifically examine the effects of cold IRI, while excluding the complex interaction with alloimmune responses. Based on this study, further studies should elucidate the impact of IL‐2C treatment on cold IRI in the context of allogeneic kidney transplantation.

Nevertheless, the present study elucidated the protective roles of Tregs against renal cold IRI after kidney transplantation and the protective effects of IL‐2C treatment, which represents a powerful and convenient Treg inducer, on renal cold IRI. We believe that our findings considerably contribute to the transplantation field by establishing a basis for studying the effects of immunosuppressive cells on renal cold IRI and proposing the potential utility of IL‐2C treatment in human renal cold IRI. In respect to safety, IL‐2C therapy induced little adverse effects and suppressed systemic levels of proinflammatory cytokines, as previous studies showed.^28^, ^55^ We anticipate that Treg‐inducing humanized anti‐IL‐2 monoclonal antibodies, such as F5111.2, will be successfully developed for future application of IL‐2C.^56^

In conclusion, Tregs have a protective role against cold IRI after kidney transplantation. Treatment with IL‐2C reduced acute renal injury, promoted subacute renal regeneration and inhibited chronic fibrosis in cold IRI by increasing the Treg population in kidneys, suggesting a potential of Tregs and IL‐2C in the treatment of renal cold IRI.

## AUTHOR CONTRIBUTIONS

Hyung Woo Kim and Jaeseok Yang designed the study; Joon Young Jang, Ji‐Jing Yan, Tae Kyeom Kang and Wook‐Bin Lee performed the experiments; Joon Young Jang, Hyung Woo Kim, Ji‐Jing Yan, Beom Seok Kim and Jaeseok Yang analyzed the data; Hyung Woo Kim and Jaeseok Yang wrote the manuscript; all authors approved the final version of the manuscript.

## CONFLICT OF INTEREST STATEMENT

The authors declare no conflict of interest.

## FUNDING INFORMATION

This study was supported by grants from the Ministry of Science and ICT (NRF‐2018R1A2B3001179 and NRF‐2022R1A2C3003496), which were not involved in the design or analysis of the study.

## ETHICS STATEMENT

All experiments were approved by the Institutional Animal Care and Use Committee of the Yonsei University Health System (IACUC 2021−0131) and adhered to the NIH Guide for the Care and Use of Laboratory Animals or the equivalent.

## Supporting information

Supporting Information
Table S1 Materials used in the present study.
Table S2 Primer sets used for real‐time reverse transcription‐polymerase chain reaction.
Figure S1 Flow cytometric gating strategies for renal immune cells.
Figure S2 Full images of western blots.
Figure S3 Cold IRI with a long CIT induced more severe renal infiltration of inflammatory cells and renal expression of fibrosis‐related molecules.
Figure S4 IL‐2C‐treated Tregs suppressed in vitro T cell proliferation in a dose‐dependent manner.
Figure S5 Adoptive transfer of IL‐2C‐untreated Tregs attenuated renal cold IRI.
Figure S6 Immunofluorescence images showing renal infiltration of macrophages and neutrophils on day 1 after cold IRI.
Figure S7 IL‐2C treatment induced the expansion of renal and splenic Tregs after cold IRI.
Figure S8 Impact of IL‐2C therapy on renal innate immune cells after cold IRI.
Figure S9 IL‐2C therapy increased renal tubular expression of AQP‐1 and VEGF after cold IRI.
Figure S10 IL‐2C therapy reduced renal α‐SMA expression and increased renal E‐cadherin expression after cold IRI.
Figure S11 IL‐2C treatment suppressed ROS generation and enhanced antioxidant function in cold IRI.
Figure S12 IL‐2C therapy suppressed systemic inflammation after renal IRI.

## Data Availability

All data are included in the manuscript or supporting materials.
