# Supplementary material for: Interleukin‐2/anti‐interleukin‐2 immune complex attenuates cold ischemia‐reperfusion injury after kidney transplantation by increasing renal regulatory T cells
Source: Clin Transl Med. 2024 Mar 19;14(3):e1631. doi: 10.1002/ctm2.1631 (PMC10951489; doi:10.1002/ctm2.1631)
Supplement: Supplementary file 1 — Supporting Information Table S1 Materials used in the present study. Table S2 Primer sets used for real‐time reverse transcription‐polymerase chain reaction. Figure S1 Flow cytometric gating strategies for renal immune cells. Figure S2 Full images of western blots. Figure S3 Cold IRI with a long CIT induced more severe renal infiltration of inflammatory cells and renal expression of fibrosis‐related molecules. Figure S4 IL‐2C‐treated Tregs suppressed in vitro T cell proliferation in a dose‐dependent manner. Figure S5 Adoptive transfer of IL‐2C‐untreated Tregs attenuated renal cold IRI. Figure S6 Immunofluorescence images showing renal infiltration of macrophages and neutrophils on day 1 after cold IRI. Figure S7 IL‐2C treatment induced the expansion of renal and splenic Tregs after cold IRI. Figure S8 Impact of IL‐2C therapy on renal innate immune cells after cold IRI. Figure S9 IL‐2C therapy increased renal tubular expression of AQP‐1 and VEGF after cold IRI. Figure S10 IL‐2C therapy reduced renal α‐SMA expression and increased renal E‐cadherin expression after cold IRI. Figure S11 IL‐2C treatment suppressed ROS generation and enhanced antioxidant function in cold IRI. Figure S12 IL‐2C therapy suppressed systemic inflammation after renal IRI. [file CTM2-14-e1631-s001.docx]

**SUPPLEMENTAL MATERIALS**

**SUPPLEMENTAL METHODS**

**Animals**

C57BL/6J (B6) mice and forkhead box P3 (Foxp3)-green fluorescent protein (GFP)-diphtheria toxin receptor (DTR) B6 mice were purchased from Japan SLC Inc. (Hamamatsu, Japan) and the Jackson Laboratory (Bar Harbor, ME, USA), respectively. Foxp3-knock-in (KI) B6 mice were provided by Dr. A.Y. Rudensky (Memorial Sloan Kettering Cancer Center, New York City, NY, USA). All experimental animals were maintained in specific pathogen-free animal facilities. All animals were housed and received humane care in compliance with the Principles of Laboratory Animal Care as formulated by the National Society for Medical Research. Tramadol (0.25 mg/kg; Jeil Pharmaceutical, Seoul, Republic of Korea) was subcutaneously administrated immediately after kidney transplantation to relieve pain.

**Flow cytometric analysis**

Isolated cells were stained with antibodies against CD45, CD3, CD4, Gr-1, F4/80, CD11b, Ly6C, CD25, Foxp3, NK1.1, Lin, CD127, CD90, IL-10, and 7-aminoactinomycin D (Table S1). For intracellular staining, the cells were fixed and permeabilized using Foxp3/Transcription Factor Staining Buffer Set (Thermo Fisher Scientific, Waltham, MA, USA), and live cells were stained using LIVE/DEAD Fixable Dead Cell Stain Kit (Thermo Fisher Scientific). For intracellular IL-10 staining, kidney cells were incubated with 500 ng/ml of ionomycin (Merk, Pahway, NJ, USA) and 50 ng/ml of phorbol myristate acetate and GolgiStop (BD Biosciences) at 37ºC for 5 hours. Flow cytometric analysis was performed using an Attune NxT flow cytometer (Thermo Fisher Scientific) and FlowJo software (Tree Star, Ashland, OR, USA).

***In vitro* suppression assay**

CD4^+^Foxp3 (GFP)^+^ Treg cells and CD4^+^Foxp3 (GFP)^-^ effector T cells were sorted from IL-2C-treated and IL-2C untreated Foxp3-GFP mice, respectively. Fixed number of effector T cells (2×10^5^/well) were co-cultured with Tregs at various ratios and stimulated with plate-bound anti-CD3 and anti-CD28 antibodies (5 μg/ml; Biolegend) for 3 days. T cell proliferation was assessed by thymidine uptake, and the degree of proliferation was expressed as relative proliferation (%) compared to proliferation activity in the effector T cell alone group.

**Measurement of 8-hydroxy-2'-deoxyguanosine, malondialdehyde, and glutathione levels and determination of superoxide dismutase activity**

The 8-OHdG and MDA levels were measured using an enzyme-linked immunosorbent assay kit (MyBioSource Inc., San Diego, CA, USA). GSH levels were measured using a GSH colorimetric assay kit (Abcam, Cambridge, United Kingdom). SOD activity was determined using the SOD Activity Assay kit (Cayman Chemical, Ann Arbor, MI, USA).

**Real-time polymerase chain reaction**

Renal tissues were homogenized in Trizol reagent (Thermo Fisher Scientific), and cDNA was synthesized using Superscript II Reverse Transcriptase (Thermo Fisher Scientific) with oligo-dT as the primers. The expression level of individual genes was measured using a QuantStudio 3 Real-Time PCR system (Thermo Fisher Scientific). The mRNA level of each gene was normalized to that of glyceraldehyde 3-phosphate dehydrogenase.

**Western blotting**

Renal tissues were lysed using radioimmunoprecipitation assay lysis buffer containing complete protease-inhibitor cocktail tablets (Roche, Basel, Switzerland). Signals developed using Amersham ECL reagents were detected using the ImageQuant LAS 4000 system (GE Healthcare, Chicago, IL, USA). The ImageJ plugin (https://imagej.nih.gov) was used for densitometry calculation. All antibodies were used in 1:1000 dilution conditions for western blot analysis.

**Histological analysis**

Kidney sections from 4% paraformaldehyde-fixed and paraffin-embedded tissues were stained with PAS, MT, Ki67, α-SMA, E-cadherin, Aquaporin-1 (AQP1), vascular endothelial growth factor (VEGF), and TUNEL in immunohistochemical staining. Five HPFs (×200) were captured and the mean number of positive cells in the field was calculated for quantification. The tubular injury score was assessed in periodic acid–Schiff (PAS)-stained sections based on morphological variables, including tubular dilatation, tubular cell necrosis, cast formation, and tubular brush-borders. The tubular injury score was graded from 1 to 4 by assessing the percentage of affected tubules (0: normal; 1: 1–25%; 2: 26–50%; 3: 50–75%; and 4: 75–100%) in at least four randomly selected high-power fields (HPFs) in each section. Renal fibrosis in MT staining was measured as proportions of the blue-stained area in the renal cortex and outer medulla using ImageJ software. Renal cortical thickness was also measured using ImageJ software. All histological analyses were performed by two independent researchers blinded to the treatment group. For IF staining, kidney cryosections were stained with anti-F4/80, anti-Gr-1, anti-CD4 (Alexa Flour 488) and anti-Foxp3 (eFlour 570) antibodies, and cell nuclei were counterstained with 4′,6-diamidino-2-phenylindole. The cell permeable fluorescent dye dihydroethidium (DHE) was used to detect intracellular reactive oxygen species (ROS). The frozen kidney sections were incubated with 10 μM DHE for 30 min in a dark and humidified room at room temperature and then counterstained with 4′,6-diamidino-2-phenylindole (DAPI). Images were observed using a Leica TCS SP8 confocal laser-scanning microscope (Leica, Wetzlar, Germany) at ×400 magnification. The fluorescence intensities were quantified using ZEN 2012 Software (Zeiss).

**Table S1**. **Materials used in the present study**

| **Reagents** | | **Identifier** | | | **Source (company)** |
| --- | --- | --- | --- | --- | --- |
| Antibodies | | | | | |
| anti-Aquaporin 1 | Cat# AB2219, polyclinal, Use: IHC | | | Merck | |
| anti-α-SMA | Cat#ab124964, Clone: EPR5368, Use: WB, IHC | | | Abcam | |
| anti-α-SMA-FITC | Cat#53-9760, Clone:1A4, Use: FACS | | | Thermo Fisher Scientific | |
| anti-β-Actin | Cat#ab115777, Clone: SP124 Use: WB | | | Abcam | |
| anti-CD11b-PE | Cat#553311, Clone: M1/70, Use: FACS | | | BD Biosciences | |
| anti-CD127-PE | Cat#12-1271, Clone: A7R34, Use: FACS | | | Thermo Fisher Scientific | |
| anti-CD25-APC-Cy7 | Cat #12-0251, Clone: PC61.5, Use: FACS | | | Thermo Fisher Scientific | |
| anti-CD3-APC | Cat#17-0032, Clone: 17A2, Use: FACS | | | Thermo Fisher Scientific | |
| anti-CD3 | Cat#100238, Clone: 17A2, Use: suppression assay | | | BioLegend | |
| anti-CD4-PE-Cy7 | Cat#25-0041, Clone: GK1.5, Use: FACS | | | Thermo Fisher Scientific | |
| anti-CD4-Alexa Fluor 488 | Cat#100529, Clone: RM4-5, Use: IF | | | BioLegend | |
| anti-CD28 | Cat#102116, Clone:37.51, Use: suppression assay | | | BioLegend | |
| anti-CD45-V421 | Cat#103134, Clone: 30-F11, Use: FACS | | | BioLegend | |
| Anti-CD90-APC-cy7 | Cat#47-0903, Clone: 30-H12, Use: FACS | | | Thermo Fisher Scientific | |
| anti-Col-IV | Cat#ab6586, polyclonal, Use: WB | | | Abcam | |
| anti-E-cadherin | Cat#3195, Clone: 24E10, Use: WB, IHC | | | Cell signaling | |
| anti-F4/80-FITC | Cat#11-4801, Clone: BM8, Use: FACS | | | Thermo Fisher Scientific | |
| anti-F4/80-Alexa Fluor 488 | Cat# 123120, Clone: BM8, Use: IF | | | Biolegend | |
| anti-Fibronectin | Cat#ab45688, Clone: F14, Use: WB | | | Abcam | |
| anti-Foxp3-PE | Cat#11-5773, Clone: FJK-16s, Use: FACS | | | Thermo Fisher Scientific | |
| anti-Foxp3-eFluor 570 | Cat#41-5773, Clone: FJK-16s, Use: IF | | | Thermo Fisher Scientific | |
| anti-Gr-1-APC | Cat#17-5931, Clone: RB6-8C5, Use: FACS | | | Thermo Fisher Scientific | |
| anti-Gr-1 | Cat#14-5931, Clone: RB6-8C5, Use: IF | | | Thermo Fisher Scientific | |
| anti-IL10-V421 | Cat#505022, Clone: JES5-16E3 | | | Biolegend | |
| anti-Ki67 | Cat#ab16667, Clone: SP6, Use: IHC | | | Abcam | |
| anti-Ly6C-APC-Cy7 | Cat#560596, Clone: AL-21, Use: FACS | | | BD Biosciences | |
| anti-mouse lineage (Lin) | Cat#22-7770, Use: FACS | | | Thermo Fisher Scientific | |
| anti-Nox2 | Cat#ab129068, Clone: EPR6991, Use: WB | | | Abcam | |
| anti-TGFβ | Cat#ab215715, Clone: EPR21143, Use: WB | | | Abcam | |
| anti-VEGF | Cat#BS-0279, Polyclonal, Use: IHC | | | Bioss | |
| anti-Vimentin | Cat#5741, Clone: D21H3, Use: WB | | | Cell signaling | |
| Chemical or recombinant protein | | | | | |
| 7-AAD | | | Cat#00-6993-50, Use: FACS | | Thermo Fisher Scientific |
| LIVE/DEAD™ Fixable red dead cell stain kit | | | Cat#L34972, Use: FACS | | Thermo Fisher Scientific |
| Diphtheria Toxin (DT) | | | Cat#D0564 | | Merck |
| Anti-mouse IL-2 | | | Cat#BE0043, Clone: JES6-1 | | BioXCell |
| Mouse IL-2 | | | Cat#575408 | | BioLegend |
| Assay kits | | | | | |
| CHEM8^+^ Cartridge | | | CHEM8^+^, Detect: Creatinine, BUN | | Abbott |
| MojoSort™ CD4 T Cell Isolation Kit | | | Cat#480005 | | BioLegend |
| TUNEL assay kit | | | Cat#ab206386 | | Abcam |
| 8-OHdG ELISA kit | | | Cat#MBS163648 | | MyBioSource |
| MDA ELISA kit | | | Cat#MBS269473 | | MyBioSource |
| GSH Assay kit | | | Cat#ab239727 | | Abcam |
| SOD Assay kit | | | Cat#706002 | | Cayman |

7-AAD, 7-aminoactinomycin D; APC, allophycocyanin; α-SMA, α-smooth muscle actin; BUN, blood urea nitrogen; Col-IV, type IV collagen; ELISA, enzyme-linked immunosorbent assay; FACS, fluorescence-activated cell sorting; FITC, fluorescein; Foxp3, forkhead box P3; GSH, Glutathione; IF, immunofluorescence staining; IHC, immunohistochemical staining; IL, interleukin; IL-2C, IL-2/anti-IL-2 antibody immune complex; MDA, Malondialdehyde; Nox2, nicotinamide adenine dinucleotide phosphate oxidase 2; 8-OHdG, 8-hydroxy-2'-deoxyguanosine; PE, phycoerythrin; SOD, superoxide dismutase; TGF-β, transforming growth factor-beta; TUNEL, terminal deoxynucleotidyl transferase dUTP nick-end labeling; VEGF, vascular endothelial growth factor; WB, western blot.

**Table S2**. **Primer sets used for real-time reverse transcription-polymerase chain reaction**

| **Gene** | **Gene Bank ID** | **Primer sequence (5’-3’)** |
| --- | --- | --- |
| *Acta2* | *NM_007392.3* | F: GGCATCCACGAAACCACCTAT  R: TTCTGGAGGGGCAATGATCTTG |
| *Col4a1* | *NM_009931.2* | F: GCTCTGGCTGTGGAAAATGTG  R: GTTCTCCAGCATCACCCTTTTG |
| *Cdh1* | *NM_009864.3* | F: CAGCCGGTCTTTGAGGGATT  R: TGACGATGGTGTAGGCGATG |
| *Fn1* | *NM_010233* | F: TTGGTGATGTGTGAAGGCTC  R: ACCTCTGCAGACCTACCCAG |
| *Gapdh* | *NM_008084* | F: CAGAGGCGCATGAAGCTAATG  R: CCTGGATCTTCCTCACTTGCT |
| *Infg* | *NM_008337* | F: GAGCTCATTGAATGCTTGGC  R: GCGTCATTGAATCACACCTG |
| *Il10* | *NM_010548* | F: GGTGAGAAGCTGAAGACCCT  R: TGTCTAGGTCCTGGAGTCCA |
| *IL1b* | *NM_008361* | F: TCAACCAACAAGTGATATTCTC  R: ACACAGGACAGGTATAGATTC |
| *Mcp1* | *NM_011333* | F: TTCCACAACCACCTCAAGCACTTC  R: TTAAGGCATCACAGTCCGAGTCAC |
| *Nox2* | *NM_007807.5* | F: ACAACCCTCCCTGTCTAGGTA  R: GCCTTCGGTGATGTGCTTTAC |
| *Tgfb1* | *NM_011577.2* | F: AGGGCTACCATGCCAACTTC  R: CCACGTAGTAGACGATGGGC |
| *Tnfa* | *NM_013693* | F: ATGTCCATTCCTGAGTTCTG  R: AATCTGGAAAGGTCTGAAGG |
| *Vim* | *NM_011701.4* | F: GCCAGCAGTATGAAAGCGTG  R: ACCTGTCTCCGGTACTCGTT |

*Acta2*, α smooth muscle actin; *Cdh1,* epithelial cadherin; *Col4a1*, type IV collagen; F, forward; *Fn1*, fibronectin; *Gapdh*, **glyceraldehyde 3-phosphate dehydrogenase**; *Ifng*, interferon-γ; *Il10*, interleukin-10; *Il1b*, interleukin-1*β*; *Mcp1*, Monocyte chemoattracted protein-1; *Nox2*, nicotinamide adenine dinucleotide phosphate oxidase 2; *Tgfb1, t*ransforming growth factor *β; Tnfa*, tumor necrosis factor-alpha; R, reverse; *Vim*, vimentin.

**SUPPLEMENTAL FIGURES**

**

**

**Figure S1. Flow cytometric gating strategies for renal immune cells.**

(A) F4/80^+^CD11b^+^ macrophages, Gr-1^+^CD11b^+^ neutrophils, CD3^+^ T cells, and NK1.1^+^CD3^-^ NK cells were gated from singlet live CD45^+^ cells. (B) Foxp3^+^CD4^+^ regulatory T cells, Lin^-^CD127^+^ ILCs, and Lin^-^CD127^+^CD90^+^IL-10^+^ ILCregs were gated from singlet live CD45^+^ cells. 7-AAD, 7-aminoactinomycin D; Foxp3, forkhead box P3; FSC, forward scatter; ILCs, innate lymphoid cells; ILCregs, regulatory innate lymphoid cells; NK, natural killer; SSC, side scatter.

**

**

**Figure S2. Full images of western blots.**

(A) Full western blot images for Figure 6B (TGF-β, α-SMA, fibronectin, type IV collagen, β-actin). (B) Full western blot images for Figure 6F (E-cadherin, vimentin, β-actin). (C) Full western blot images for Figure 7C (Nox2, β-actin). α-SMA, α-smooth muscle actin; Col-IV, type IV collagen; Nox2, nicotinamide adenine dinucleotide phosphate oxidase; TGF-β, transforming growth factor-beta.

**
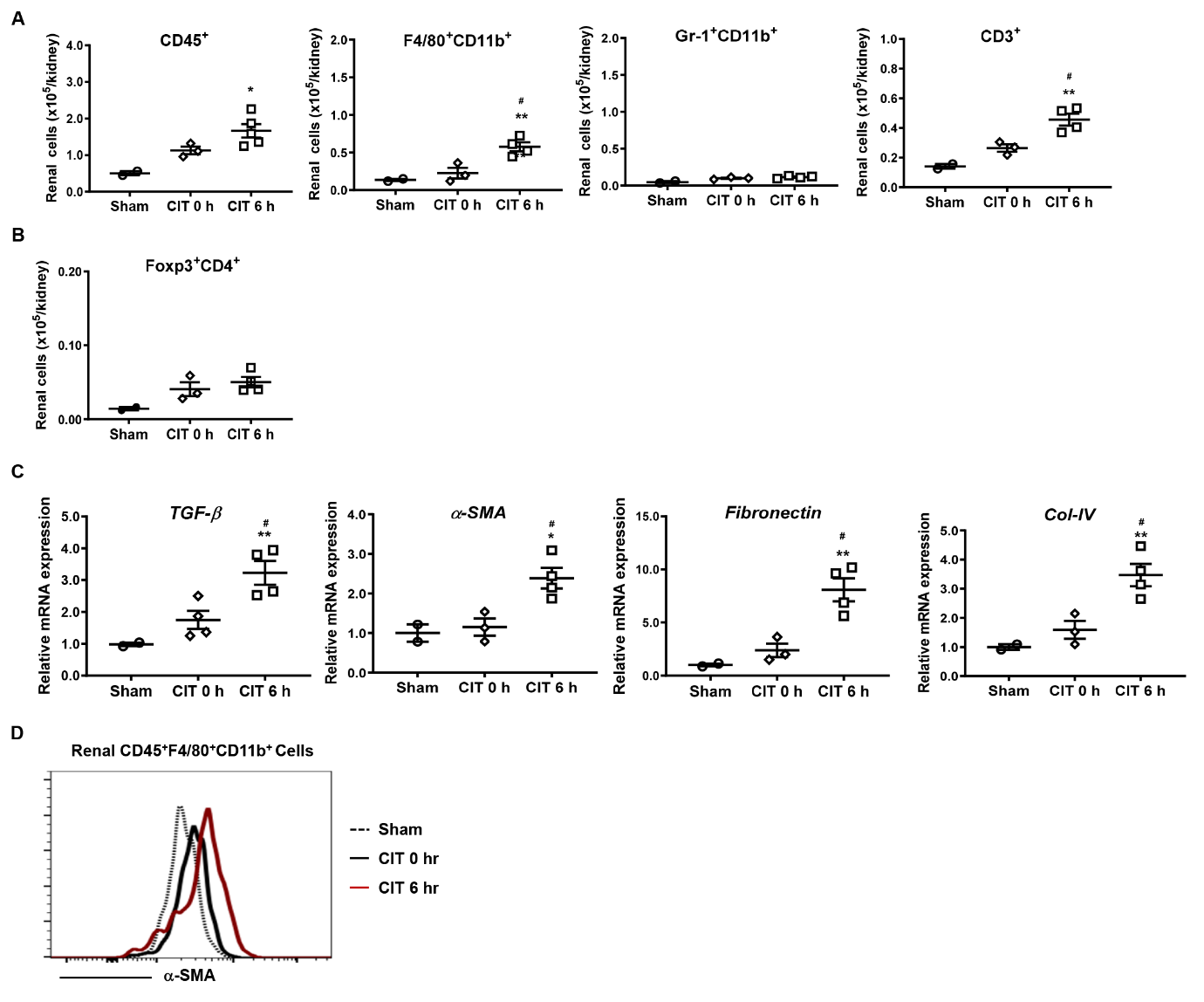
**

**Figure S3.** **Cold IRI with a long CIT induced more severe renal infiltration of inflammatory cells and renal expression of fibrosis-related molecules.**

(A) Absolute numbers of renal CD45^+^, F4/80^+^CD11b^+^, Gr-1^+^CD11b^+^, and CD3^+^ cells. (B) Absolute numbers of renal CD4^+^Foxp3^+^ Tregs on day 28 after cold IRI. (C) Renal mRNA expression level of *TGF-β* (*Tgfb1)*, *α-SMA* (*Acta2*), *fibronectin (Fn1)*, and *Col-IV (Col4a1)* normalized to *Gapdh* expression level on day 28 after cold IRI. (D) Expression of α-SMA in renal F4/80^+^CD11b^+^ macrophages. Lines and whiskers in dot plots indicate the mean and SEM, respectively. *P < 0.05, **P < 0.01 compared with sham group; ^#^P < 0.05, ^##^P < 0.01 compared with IRI with CIT of 0 h. α-SMA, α-smooth muscle actin; CIT, cold ischemic time; Col-IV, type IV collagen; *Gapdh*, **glyceraldehyde 3-phosphate dehydrogenase**; SEM, standard error of the mean; TGF-β, transforming growth factor-β.

**

**

**Figure S4.** **IL-2C-treated Tregs suppressed *in vitro* T cell proliferation in a dose-dependent manner.**

CD4^+^Foxp3 (GFP)^-^ effector T cells isolated from IL-2C-untreated Foxp3-GFP knockin mice were cocultured with CD4^+^Foxp3 (GFP)+ Tregs isolated from IL-2C-treated Foxp3-GFP knockin mice at different ratios. Proliferation in response to anti-CD3/CD28 stimulation was expressed as relative proliferation (%) compared to proliferation of the effector T cell alone group. Lines and whiskers in dot plots indicate the mean and SEM, respectively. *P < 0.05, **P < 0.01 compared with the effector T cell alone group. Foxp3, forkhead box P3; GFP, green fluorescent protein; IL-2C, interleukin-2/anti-IL-2 antibody immune complex; SEM, standard error of the mean; Teffs, effector T cells; Tregs, regulatory T cells.

**
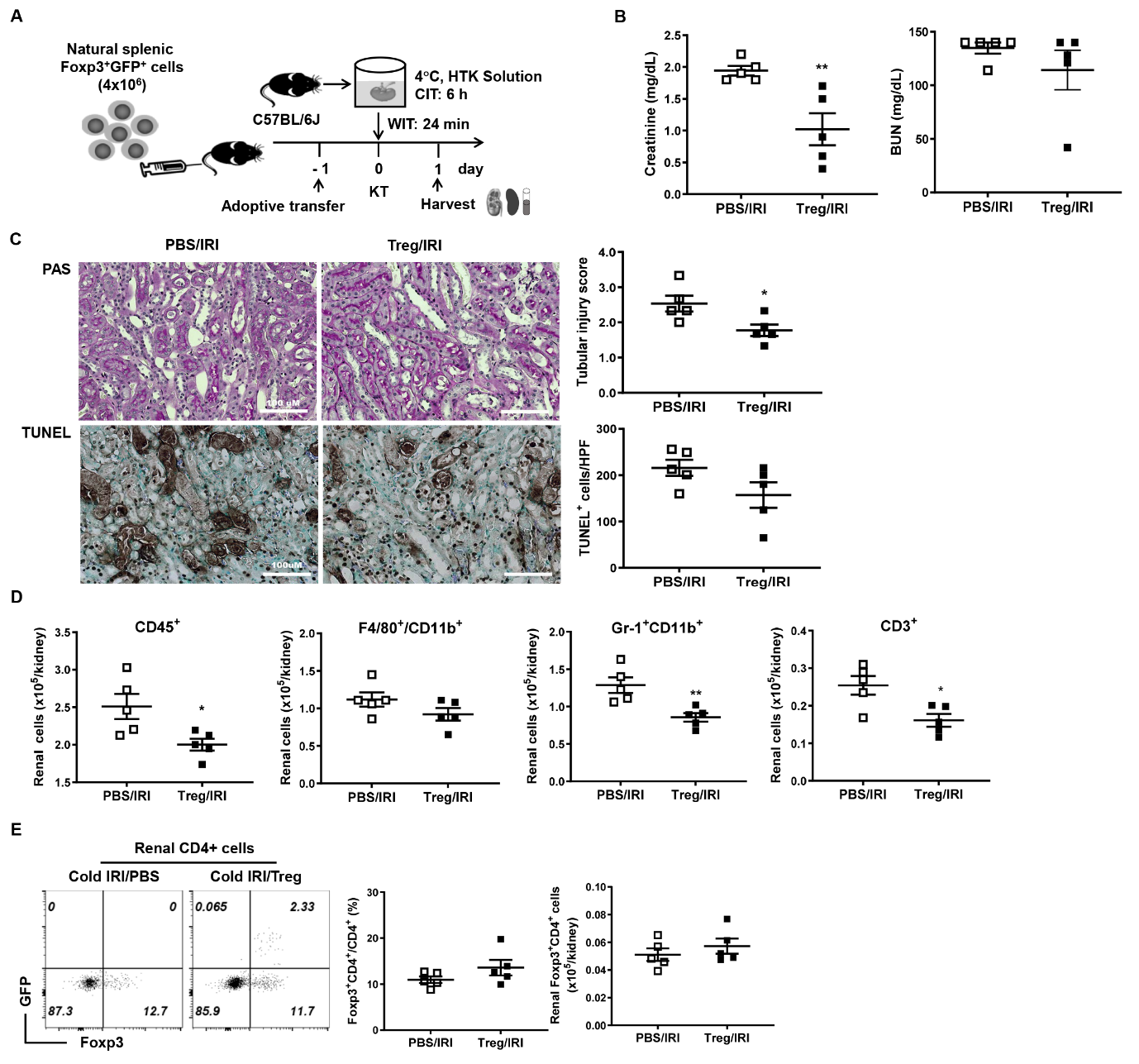
**

**Figure S5. Adoptive transfer of IL-2C-untreated Tregs attenuated renal cold IRI.**

(A) Natural CD4^+^Foxp3^+^ Tregs without IL-2C treatment were adoptively transferred to recipient mice 1 d before inducing cold IRI and kidneys were harvested along with blood sampling on day 1. (B) Level of blood creatinine and BUN. (C) Renal tissue injury scores and renal tubular apoptosis (based on TUNEL staining). Magnification, 200×. (D) Absolute numbers of renal CD45^+^, F4/80^+^CD11b^+^, Gr-1^+^CD11b^+^, and CD3^+^ cells. (E) Proportions and absolute number of renal CD4^+^Foxp3^+^ Tregs. Lines and whiskers in dot plots indicate the mean and SEM, respectively. *P < 0.05, **P < 0.01 compared with PBS/IRI group. BUN, blood urea nitrogen; CIT, cold ischemic time; Foxp3, forkhead box P3; GFP, green fluorescent protein; HPF, high-power field; HTK, histidine-tryptophan-ketoglutarate; IL-2C, interleukin-2/anti-IL-2 antibody immune complex; IRI, ischemia-reperfusion injury; KT, kidney transplantation; PAS, periodic acid–Schiff; PBS, phosphate-buffered saline; SEM, standard error of the mean; Tregs, regulatory T cells; TUNEL, terminal deoxynucleotidyl transferase dUTP nick-end labeling; WIT, warm ischemic time.

**
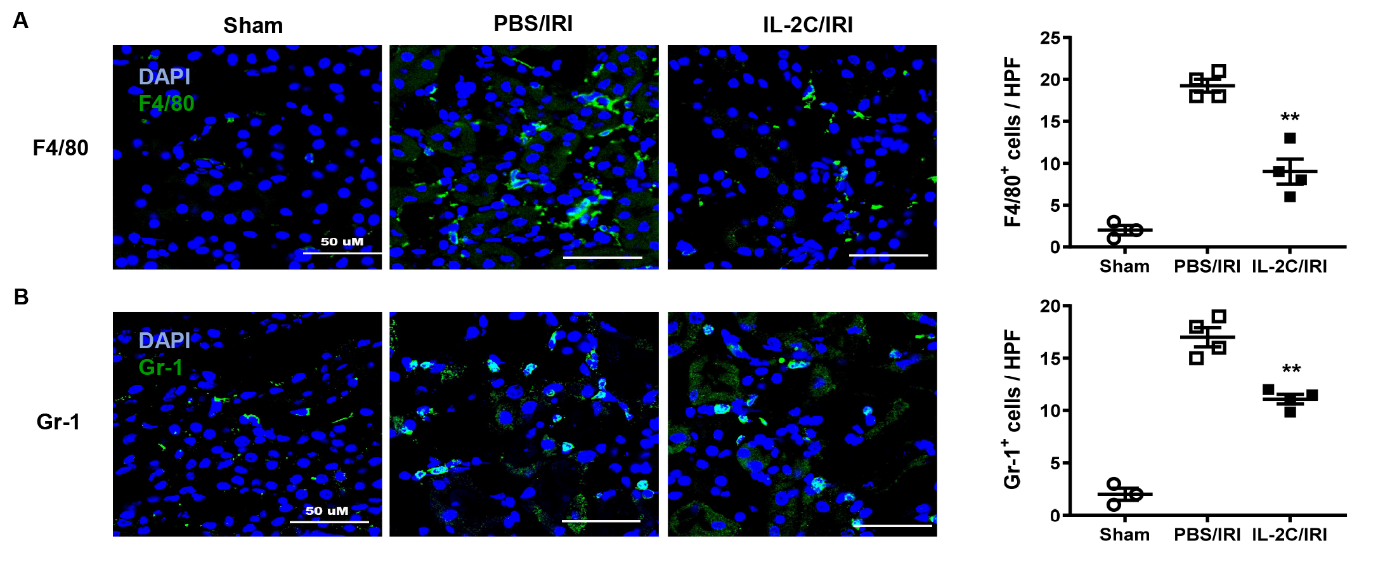
**

**Figure S6. Immunofluorescence images showing renal infiltration of macrophages and neutrophils on day 1 after cold IRI.**

(A) Green and blue color indicate DAPI and F4/80, respectively. (B) Green and blue color indicate DAPI and Gr-1, respectively. Magnification, 400×. Lines and whiskers in dot plots indicate the mean and SEM, respectively. *P < 0.05, **P < 0.01 for IL-2C/IRI groups vs. PBS/IRI groups. DAPI, 4′,6-diamidino-2-phenylindole; HPF, high power field; IL-2C, IL-2/anti-IL-2 antibody immune complex; IRI, ischemia-reperfusion injury; PBS, phosphate-buffered saline; SEM, standard error of the mean.

**
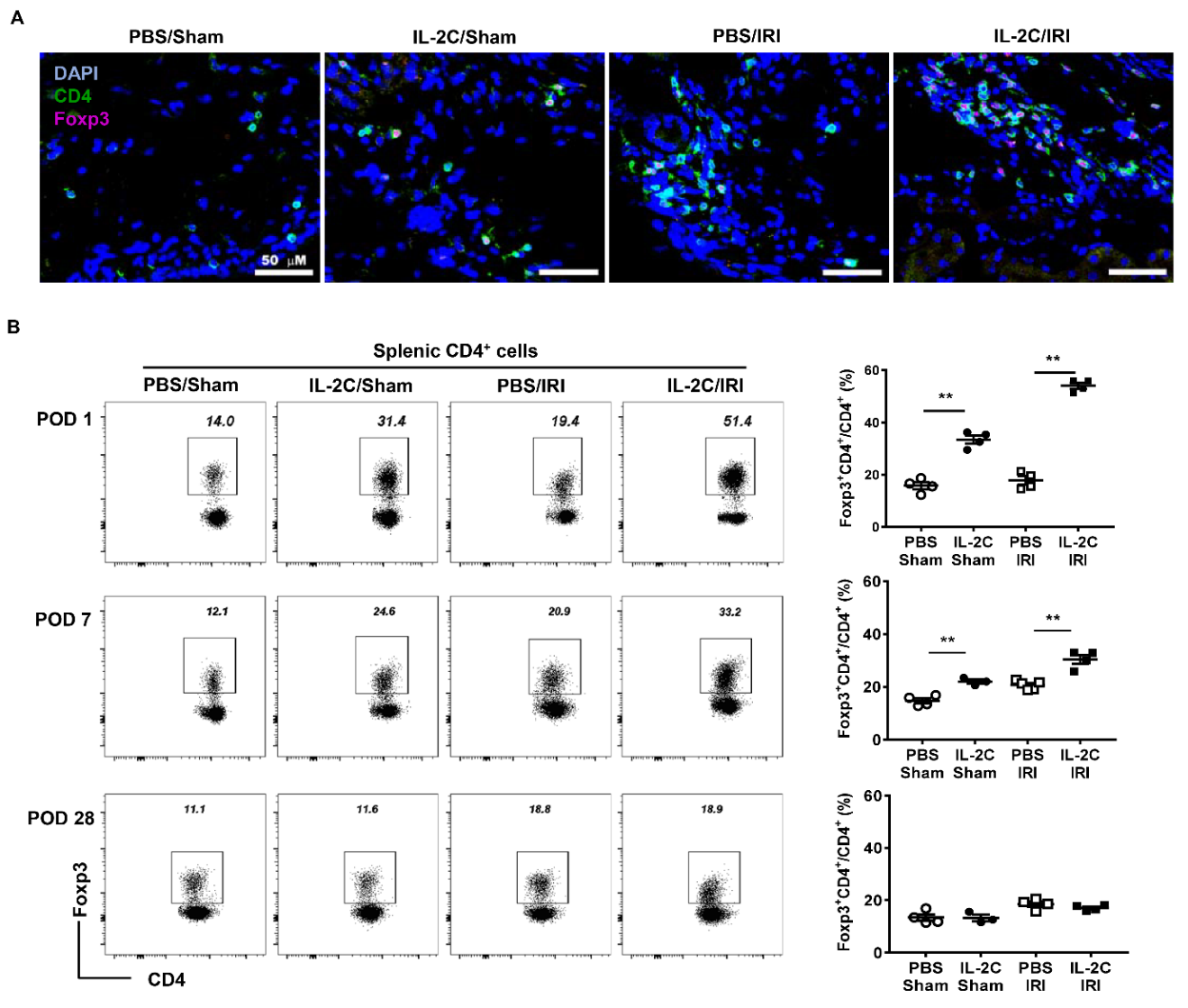
**

**Figure S7. IL-2C treatment induced the expansion of renal and splenic Tregs after cold IRI.**

(A) Immunofluorescence images showing renal infiltration of CD4^+^Foxp3^+^ Tregs on day 1 after cold IRI. Green, pink, and blue color indicate CD4, Foxp3, and DAPI, respectively. Magnification, 400×. (B) Flow cytometric diagrams for splenic CD4^+^Foxp3^+^ Tregs on day 1, 7 and 28 after cold IRI or sham operation, where IL-2C or PBS was administered. Proportions of splenic CD4^+^Foxp3^+^ Tregs were also shown. Lines and whiskers in dot plots indicate the mean and SEM, respectively. *P < 0.05, **P < 0.01 for IL-2C groups vs. PBS groups. DAPI, 4′,6-diamidino-2-phenylindole; Foxp3, forkhead box P3; IL-2C, IL-2/anti-IL-2 antibody immune complex; IRI, ischemia-reperfusion injury; PBS, phosphate-buffered saline; SEM, standard error of the mean; Treg, regulatory T cell.





**Figure S8. Impact of IL-2C treatment on renal innate immune cells after cold IRI.**

Absolute numbers of renal NK1.1^+^CD3^-^ NK cells, Lin^-^CD127^+^ ILCs, and Lin^-^CD127^+^CD90^+^IL-10^+^ ILCregs on day 1 after cold IRI. Lines and whiskers in dot plots indicate the mean and SEM, respectively. *P < 0.05, **P < 0.01 for PBS/IRI group vs. IL-2C/IRI group. IL-2C, IL-2/anti-IL-2 antibody immune complex; IL-10, interleukin-10; ILCs, innate lymphoid cells; ILCregs, regulatory innate lymphoid cells; IRI, ischemia-reperfusion injury; Lin, lineage; NK, natural killer; PBS, phosphate-buffered saline; SEM, standard error of the mean.


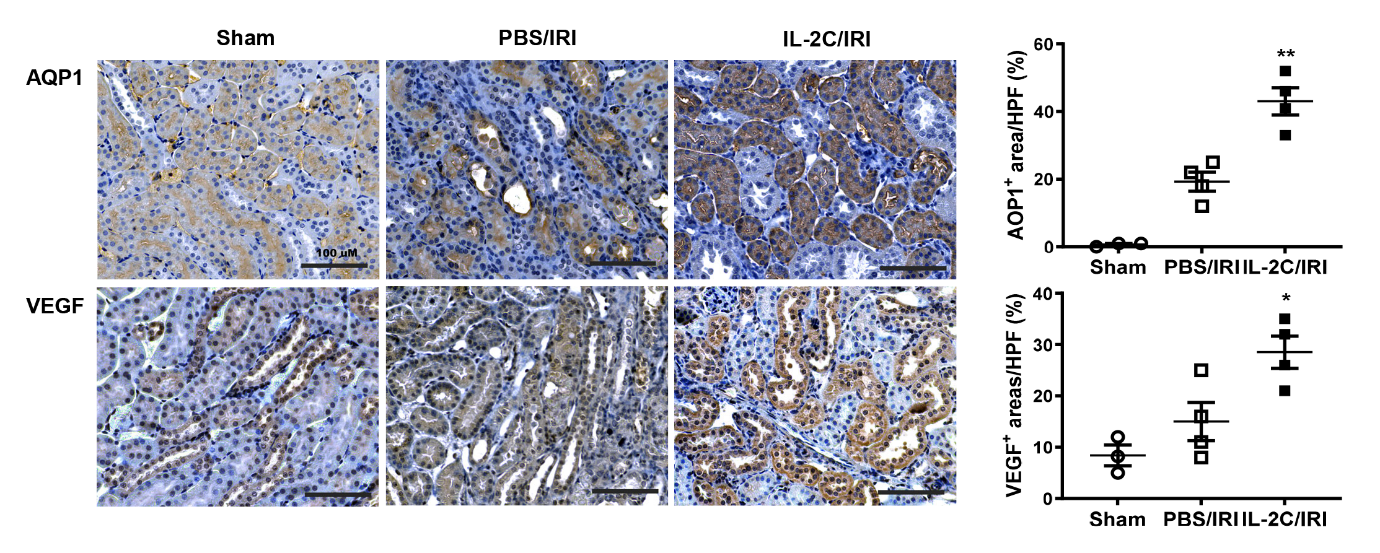


**Figure S9. IL-2C treatment increased renal tubular expression of AQP-1 and VEGF after cold IRI.**

IL-2C or PBS was administered to recipient mice seven times up to day 2 after cold IRI. Kidneys were harvested on day 7 after cold IRI. Immunohistochemical staining results for renal AQP-1 and VEGF. Magnification, 200×. Lines and whiskers in dot plots indicate the mean and SEM. *P < 0.05, **P < 0.01 for IL-2C/IRI group vs. PBS/IRI group. AQP-1, aquaporin-1; HPF, high-power field; IL-2C, IL-2/anti-IL-2 antibody immune complex; IRI, ischemia-reperfusion injury; PBS, phosphate-buffered saline; SEM, standard error of the mean; VEGF, vascular endothelial growth factor.


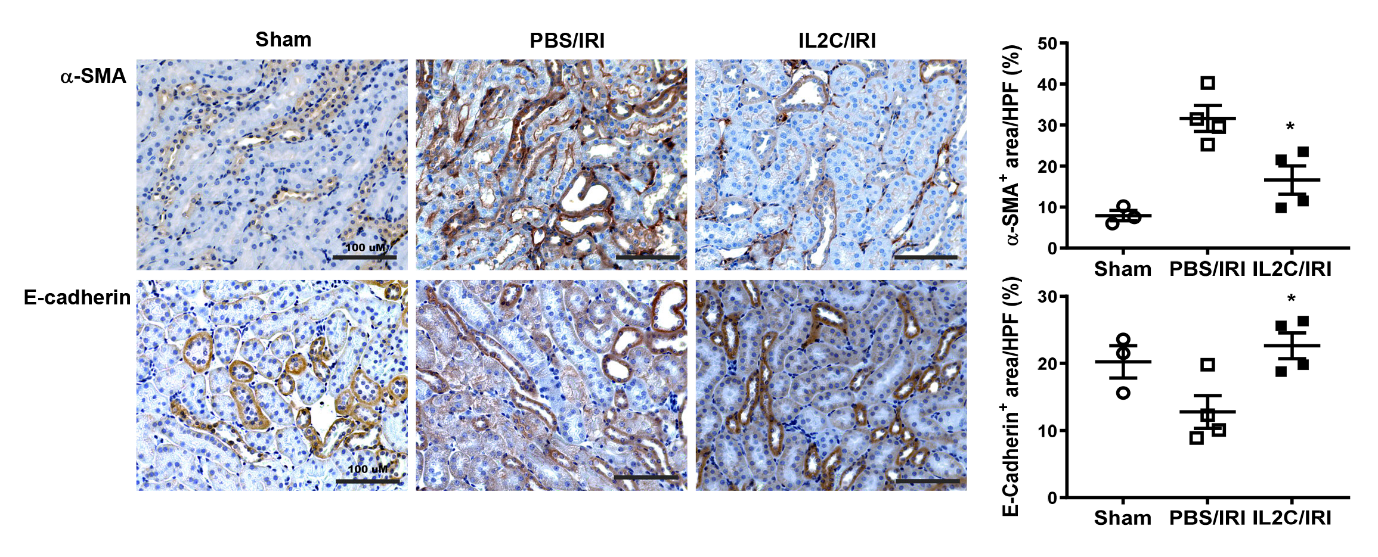


**Figure S10. IL-2C treatment decreased renal α-SMA expression and increased renal E-cadherin expression after cold IRI.**

IL-2C or PBS was administered to recipient mice for five consecutive days before inducing cold IRI and thrice a week up to 2 weeks after cold IRI. Kidneys were harvested on day 28 after cold IRI. Immunohistochemical staining results for renal α-SMA and E-cadherin. Magnification, 200×. Lines and whiskers in dot plots indicate the mean and SEM. *P < 0.05, **P < 0.01 for IL-2C/IRI group vs. PBS/IRI group. α-SMA, α smooth muscle actin; HPF, high-power field; IL-2C, IL-2/anti-IL-2 antibody immune complex; IRI, ischemia-reperfusion injury; PBS, phosphate-buffered saline; SEM, standard error of the mean; VEGF, vascular endothelial growth factor.


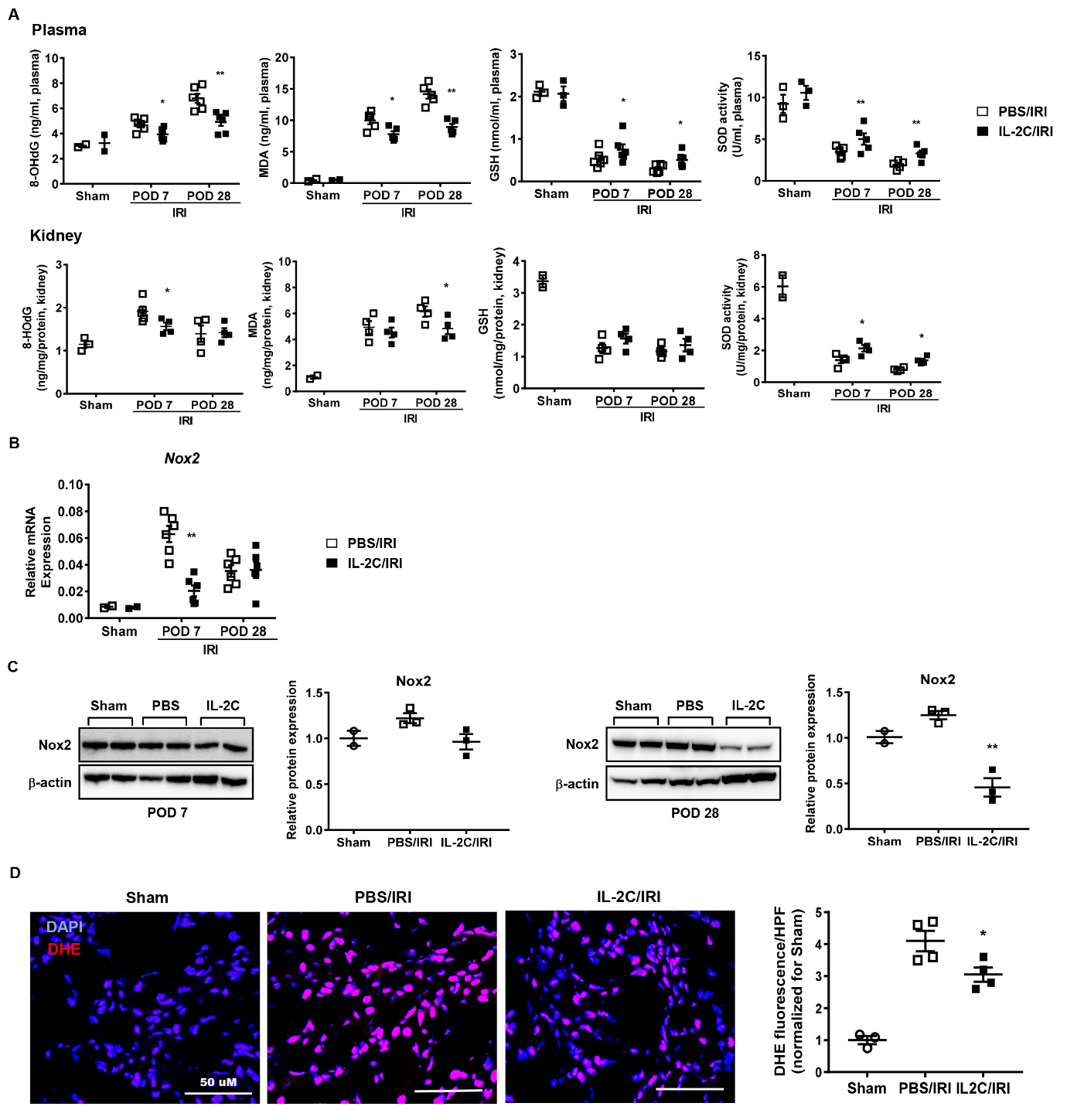


**Figure S11. IL-2C treatment suppressed ROS generation and enhanced antioxidant function in cold IRI.**

(A) Plasma and renal levels of 8-OHdG, MDA, and GSH, and SOD activity on day 7 and 28 after inducing cold IRI. (B) Renal mRNA expression level of *Nox2* normalized to *Gapdh* expression level on day 7 and 28. (C) Renal deposition of Nox2 on day 28 was measured via western blotting and normalized to β-actin levels. Lines and whiskers in dot plots indicate the mean and SEM, respectively. (D) Renal DHE staining on day 7. Pink and blue color indicate DHE and DAPI, respectively. Magnification, 400×. *P < 0.05, **P < 0.01 for IL-2C/IRI group vs. PBS/IRI group. DAPI, 4′,6-diamidino-2-phenylindole; DHE, dihydroethidium; *Gapdh*, **glyceraldehyde 3-phosphate dehydrogenase**; GSH, glutathione; HPF, high power field; IL-2C, IL-2/anti-IL-2 antibody immune complex; IRI, ischemia-reperfusion injury; MDA, malondialdehyde; Nox2, nicotinamide adenine dinucleotide phosphate oxidase 2; 8-OHdG, 8-hydroxy-2'-deoxyguanosine; PBS, phosphate-buffered saline; ROS, reactive oxygen species; SEM, standard error of the mean; SOD, superoxide dismutase.





**Figure S12. IL-2C treatment suppressed systemic inflammation after renal IRI.**

Systemic levels of IL-1β, TNF-α, IL-6, and IFN-γ on day 1 and day 28 after renal cold IRI. Lines and whiskers in dot plots indicate the mean and SEM. *P < 0.05, **P < 0.01 for PBS/IRI group vs. IL-2C/IRI group. IL-2C, IL-2/anti-IL-2 antibody immune complex; IFN-γ, interferon-γ; IL, interleukin; IRI, ischemia-reperfusion injury; PBS, phosphate-buffered saline; POD, post-operative day; SEM, standard error of the mean; TNF-α, tumor necrosis factor-α.
